# Synthesis and Anti-Melanoma Activity of L-Cysteine-Coated Iron Oxide Nanoparticles Loaded with Doxorubicin

**DOI:** 10.3390/nano13040621

**Published:** 2023-02-04

**Authors:** Luiza Izabela Toderascu, Livia Elena Sima, Stefana Orobeti, Paula Ecaterina Florian, Madalina Icriverzi, Valentin-Adrian Maraloiu, Cezar Comanescu, Nicusor Iacob, Victor Kuncser, Iulia Antohe, Gianina Popescu-Pelin, George Stanciu, Petre Ionita, Cristian N. Mihailescu, Gabriel Socol

**Affiliations:** 1 National Institute for Laser, Plasma and Radiation Physics, 077125 Magurele, Ilfov, Romania; 2 Faculty of Chemistry, University of Bucharest, 050663 Bucharest, Romania; 3 Institute of Biochemistry of the Romanian Academy, 060031 Bucharest, Romania; 4 National Institute of Materials Physics, 077125 Magurele, Ilfov, Romania; 5 Faculty of Physics, University of Bucharest, 077125 Magurele, Ilfov, Romania

**Keywords:** magnetic nanoparticles, melanoma, L-Cys, Dox, chemotherapeutic

## Abstract

In this study, we report on the synthesis of L-Cysteine (L-Cys)-coated magnetic iron oxide nanoparticles (NPs) loaded with doxorubicin (Dox). The Fe_3_O_4_-L-Cys-Dox NPs were extensively characterized for their compositional and morpho-structural features using EDS, SAED, XRD, FTIR and TEM. XPS, Mössbauer spectroscopy and SQUID measurements were also performed to determine the electronic and magnetic properties of the Fe_3_O_4_-L-Cys-Dox nanoparticles. Moreover, by means of a FO-SPR sensor, we evidenced and confirmed the binding of Dox to L-Cys. Biological tests on mouse (B16F10) and human (A375) metastatic melanoma cells evidenced the internalization of magnetic nanoparticles delivering Dox. Half maximum inhibitory concentration IC50 values of Fe_3_O_4_-L-Cys-Dox were determined for both cell lines: 4.26 µg/mL for A375 and 2.74 µg/mL for B16F10, as compared to 60.74 and 98.75 µg/mL, respectively, for unloaded controls. Incubation of cells with Fe_3_O_4_-L-Cys-Dox modulated MAPK signaling pathway activity 3 h post-treatment and produced cell cycle arrest and increased apoptosis by 48 h. We show that within the first 2 h of incubation in physiological (pH = 7.4) media, ~10–15 µM Dox/h was released from a 200 µg/mL Fe_3_O_4_-L-Cys-Dox solution, as compared to double upon incubation in citrate solution (pH = 3), which resembles acidic environment conditions. Our results highlight the potential of Fe_3_O_4_-L-Cys-Dox NPs as efficient drug delivery vehicles in melanoma therapy.

## 1. Introduction

Over the past few years, the number of people who experience different types of skin cancer has increased rapidly. Based on the cell of origin, skin cancer can be divided into melanoma and non-melanoma [1]. The skin cancer treatment response is dependent on the type, size and location of the skin cancer, but also on the absence or presence of metastases.

A range of treatment options are available at this moment and include surgical interventions, chemotherapy, radiotherapy and the use of immune checkpoint inhibitors.

Non-targeted chemotherapy is no longer desired in advanced skin cancer treatment due to the extensive organ toxicity. As an alternative approach, targeted therapies are currently being developed that are able to inhibit the growth or induce the regression of a tumor and also stop the spread of cancer cells [1,2,3]. However, intrinsic resistance and overall undesired side effects remain a challenge in melanoma therapy.

The continuous progress in nanotechnology has enabled the incorporation of various diagnosis, therapeutic and targeting agents into nanoparticles (NPs) for the detection, prevention and treatment of oncologic diseases [4,5]. Further, improvement of the treatment options is desired in order to effectively treat tumors with a minimum of side effects [6,7,8]. In this respect, the nanocarrier-based platforms, with tunable properties, have the ability to enhance the biodistribution and to deliver the anti-cancer drugs into tumors in a targeted way and to leave the healthy cells unaffected [7,8,9,10].

Magnetic nanomaterials, mainly the superparamagnetic iron oxide nanoparticles (SPIONs), have attracted the attention of many fields such as biochemistry and biomedicine due to their simple synthesis methods, functionalization capability, fast magnetic response and biocompatibility [11,12,13,14,15]. The modification of the NPs’ surface, their size and shape are significant features that interfere in the interaction between the biological systems and NPs. In recent years, SPIONs have been developed as tumor cell trackers in magnetic resonance imaging (MRI) and as heating intermediates in treatments based on magnetic hyperthermia [15,16,17,18]. However, SPIONs tend to form aggregates because of their attractive forces, such as van der Waals, which cause a difficulty in stabilizing the magnetic colloidal dispersion in solutions [16,17,18]. 

One of the proposed strategies able to improve NP stability in aqueous solutions is the surface functionalization by capping with compounds that induce electrostatic repulsive forces. L-Cysteine (L-Cys) is a non-essential, water-soluble amino acid with a strong coordination tendency and three functional groups (-NH_2_, -COOH, -SH) [19,20,21,22,23,24]. The presence of -NH_2_ and -COOH groups in the structure of this amino acid not only enhances the colloidal suspension stability and biocompatibility of NPs, but also facilitates the functionalization of magnetite surface [25]. Moreover, the high reactive capacity of L-Cys due to its -SH groups makes this amino acid responsible for many biological functions in the human body, by its ability to stabilize the tridimentional structure of proteins [26]. Depending on the cells’ needs, its assimilation can be achieved through different pathways, giving rise to sulfur compounds [26,27]. Nowadays, L-Cys-SPIONs have been used in several applications, as catalysts for synthesis [28] or to remove contaminants [29] and heavy metals [30], but, as far as we know, they have not been employed in cancer treatments.

The direct binding of Dox to the iron oxide surface can be achieved through ionic interactions, whereas in the case of L-Cys functionalization, covalent bonds between -NH_2_ exposed on the magnetite surface and -OH from the Dox structure are favorable to binding stabilization. Additionally, hydrogen bonds between -SH, -NH_2_ and -OH, not only from the L-Cys, but also from Dox, increase the loading efficiency of the NPs. The influence of ionic strength and pH on the reversibility of ionic bonds results in a faster kinetic drug release than that of covalent binding [31,32]. 

In the present article, a new approach of binding Dox to iron oxide (Fe_3_O_4_) NPs by their coating with L-Cys amino acid was proposed, which improved the colloidal stability in aqueous solutions. Moreover, L-Cys-coated NPs revealed an efficient Dox delivery after their internalization into melanoma cells. 

## 2. Materials and Methods

### 2.1. Materials

Iron (II) and (III) chloride (FeCl_2_ and FeCl_3_, respectively), ammonia solution (25%), L-Cys and ethanol were purchased from Sigma-Aldrich. Dox∙HCl was acquired from AvaChem Scientific. All chemicals were used without further purification. All the aqueous solutions were prepared using deionized water produced with a TKA-GenPure Water Purification System (TKA Wasseraufbereitungssysteme GmbH, Niederelbert, Germany).

### 2.2. Nanoparticles Synthesis

Magnetite nanoparticles (Fe_3_O_4_ NPs) were synthesized using the coprecipitation method [21]. FeCl_3_ and FeCl_2_ꞏ4H_2_O solution with 2:1 molar ratio was stirred for 30 min in a round-bottom flask. Next, 30 mL of ammonia (NH_3_) solution (25%) was added dropwise with a pipette and further stirred for one hour [31]. The as-synthesized magnetic nanoparticles were separated by means of a permanent magnet and washed several times with deionized water to remove the excess of ammonia solution [31,32]. 

The functionalization of Fe_3_O_4_ NPs with L-Cys (Fe_3_O_4_-L-Cys NPs) was performed through the sonication for 30 min of 0.1 M L-Cys and 1.5 g/mL Fe_3_O_4_ NPs aqueous suspension [32].

Dox binding to Fe_3_O_4_-L-Cys NPs (Fe_3_O_4_-L-Cys-Dox NPs) was performed by continuous stirring for 24 h of 10 mg of pure drug with 130 mL ethanol and 10 mL aqueous suspension of Fe_3_O_4_-L-Cys NPs (10 mg/L). Finally, the suspension was washed several times with deionized water to remove the unbound drug from the mixture [33]. 

### 2.3. Transmission Electron Microscopy (TEM)

The structural characterization of samples was carried out using a JEOL 2100 analytical electron microscope equipped with a JEOL JED-2300T unit used for energy-dispersive X-ray spectroscopy (EDXS) analysis. It was operated at an acceleration voltage of 200 kV and the samples were observed using conventional transmission electron microscopy (CTEM) equipped with selected area electron diffraction (SAED) at magnifications ranging from 40,000 to 300,000. The powders were crushed in an agate mortar and dispersed in ethanol. A droplet of each suspension was then deposited onto a TEM Cu grid with lacey carbon support film.

### 2.4. X-ray Diffraction (XRD)

The phase structure of pure Fe_3_O_4_, Fe_3_O_4_-L-Cys and Fe_3_O_4_-L-Cys-Dox nanoparticles was identified through X-ray diffraction (XRD) measurements using a PANalytical Empyrean diffractometer, in conventional Bragg–Brentano geometry, with CuKα radiation (λ = 1.5406 Å). A step size and a scan speed of 0.01 and 2 min/step, respectively, were employed.

### 2.5. X-ray Photoelectron Spectroscopy (XPS)

The chemical composition of samples was determined using X-ray photoelectron spectroscopy (XPS). All measurements were performed using an ESCALAB Xi+ (Thermo SCIENTIFIC Surface Analysis, Waltham, MA, USA) equipped with a multichannel hemispherical electron analyzer (dual X-ray source) working with Al Kα radiation (hν = 1486.2 eV). As reference, a peak of C 1 s at 284.8 eV was used. XPS data were recorded on slightly pressed powder materials on indium beads that had been outgassed at room temperature (RT) in the pre-chamber of the setup down to a pressure of <2 × 10^−6^ Pa in order to remove the chemisorbed water from their surfaces. The surface chemical compositions and oxidation states were estimated from the XPS spectra by calculating the integral of each peak after subtraction of the “S-shaped” Shirley-type background using the appropriate experimental sensitivity factors by means of Avantage 5.978 software.

### 2.6. Magnetic Measurements

Magnetic field investigations were carried out on a superconducting quantum interference device (SQUID) magnetometer (MPMS 7T from Quantum Design, San Diego, CA, USA) working under the reciprocal space option. Hysteresis loops at 300 K and 10 K were collected in fields of up to 2 T and zero-field-cooled–field-cooled magnetization curves measured in 100 Oe were obtained in order to investigate magnetic relaxation phenomena. Moreover, ^57^Fe Mössbauer spectra were collected at 6 K to provide deeper information on the phase composition and local magnetic structure of the investigated samples. To do so, the samples were inserted into a closed-cycle Mössbauer cryostat (Janis, Woburn, MA, USA) and a spectrometer working in the constant acceleration mode with a ^57^Co (Rh) radioactive source was used.

### 2.7. Fourier Transform Infrared (FT-IR) Spectroscopy

Fourier transform infrared (FT-IR) spectroscopy was used to identify the organic components from the composition of the magnetic nanoparticles. Dried powders were ground with potassium bromide (KBr) and further examined in reflection mode using a Shimadzu IRTracer-100 Spectrophotometer (Shimadzu Europa GmbH, Duisburg, Germany). The spectra were recorded in the (5000–500) cm^−1^ wavelength range at a resolution of 4 cm^−1^ and averaged over 50 individual scans.

### 2.8. Fiber Optic-Surface Plasmon Resonance (FO-SPR) Measurements 

The fiber optic-surface plasmon resonance (FO-SPR) setup was used to monitor the interaction between L-Cys and Dox. The FO-SPR system consists of a tungsten halogen light source (AvaLight, Avantes, The Netherlands), a UV–VIS spectrophotometer (AvaSpec 2048, Avantes, The Netherlands), an interchangeable gold-coated FO-SPR sensor and an automated computer-controlled robotic arm programmed using the ColiDrive 2.2 software (Colinbus, Belgium). The white light is guided from the tungsten halogen lamp through a bifurcated FO towards the sensor’s SPR-sensitive gold area and afterwards it is reflected back to the UV−VIS spectrophotometer. Depending on the refractive index of the medium in which the fiber is submerged, the SPR spectral resonance occurs at a certain wavelength. Every binding event at the gold surface will generate a shift in the signal response. A detailed description of the FO-SPR setup can be found in our previous articles [33,34,35,36,37,38]. Here, the freshly prepared gold-coated FO-SPR sensors were first immersed in a solution of L-Cys (0.1 M in ethanol) for four hours and then immersed in Dox (0.1 mM in ethanol) for 15 min. A control was also performed to check the binding of Dox to the gold-coated FO-SPR sensor in the absence of L-Cys. 

### 2.9. Drug Release

The concentration of Dox released from the NPs was estimated through the solubilization of 10 mg of Dox-NPs in 1 mL of Milli-Q water. To assess the capacity of Dox-NPs’ release in simulated biological environments, two buffers were used: phosphate-buffered saline (PBS), pH = 7.4 (Santa Cruz), which mimics the neutral physiological environment, and citrate buffer, pH = 3, which resembles the acidic environment of the tumor microenvironment. Citrate buffer was obtained at 0.1 M through solubilization of sodium citrate (9.2 mM concentration and 357.16 g/mol molecular mass) and citric acid (90.8 mM concentration and 210.149 g/mol molecular mass). The pH was adjusted by adding 0.1 M NaOH solution. Then, 200 µL of Fe_3_O_4_-L-Cys-Dox NPs solution (10 mg/mL) was added to 10 mL of the specific buffer. Solutions were incubated at 37 °C in a Bio RS-24 rotating system (Biosan) at speed level 2 in order to reproduce the release of the drug in a dynamic environment. After 1, 2, 24 and 48 h of incubation, NP solutions were centrifuged, applying 10,500 rpm, at 4 °C for 10 min, and 1 mL of the obtained supernatant was centrifuged thereafter at 13,000 rpm for 30 s.

The fluorescence intensity of Dox released from the NP samples was measured with a NANODROP 3300 spectrofluorometer (excitation at 480 nm and emission at 593 nm), and 1 ml of the supernatant collected at each time interval was replaced with 1 mL of the fresh buffer (PBS and citrate, respectively), resuspended by vortexing and incubated until the next measurement [38]. 

The concentration of Dox released at each time interval was estimated based on the Dox calibration curve in PBS and citrate, respectively, and extrapolated from the intensity of Dox fluorescence measured from the incubated NP samples. 

### 2.10. Cell Culture

B16F10 melanoma murine cell line was maintained in RPMI 1640 medium supplemented with stable glutamine, 10% (*v*/*v*) fetal bovine serum (FBS) and 1% (*v*/*v*) penicillin/streptomycin. CCD1070SK fibroblast and melanoma A375 human cell lines were cultivated in DMEM HG (4.5 g/L glucose) (Gibco-31966) medium supplemented with 10% (*v*/*v*) fetal bovine serum (FBS) (all from Gibco^®^ by Life Technologies, Erie County, NY, USA). All cell lines were kept at 37 °C in a humidified 5% CO_2_ atmosphere. 

NPs were irradiated by UV exposure inside the cell culture hood for 30 min prior to cell treatment.

### 2.11. MTS Assay

The cell viability and proliferation were determined using a CellTiter 96^®^ AQueous One Solution Cell Proliferation Assay kit (Promega, Madison, WI, USA) according to the manufacturer’s instructions. The absorbance was recorded at 450 nm after adding the MTS reagent ([3-(4, 5-dimethylthiazol-2-yl)-5-(3-carboxymethoxyphenyl)-2-(4-sulfophenyl)-2H-tetrazolium, inner salt]) and the incubation time interval at 37 °C was optimized for each cell line. The samples were tested in triplicate, in parallel with appropriate controls (untreated cells), and the results were expressed as average values, after subtracting the background absorbance. The absorbance values are proportional to the number of metabolically active cells.

For IC50 profile determination, B16F10 and A375 cells were treated with Dox and Fe_3_O_4_-L-Cys-Dox NPs, in serial dilution, for 72 h. Curve fitting was performed using GraphPad Prism 6.0 software (GraphPad Software, LLC, San Diego, CA, USA). To characterize the response sensitivity of mouse B16F10 and human A375 melanoma cell lines to Dox, cells were incubated in the presence of a serial dilution of drug (1–0.001 µM concentration) for 72 h before the MTS assay was performed.

### 2.12. Fluorescence Microscopy

Cells grown in 12-well plates (Corning) were washed with PBS to remove unattached cells and serum proteins. Adhered cells were fixed for 15 min at RT with 4% PFA solution. Then, cells were stored at 4 °C in PBS solution until visualization using the TissueFAXSiPlus imaging system and the plates module of the TissueFAXS 3.5.5.01.29 software (Tissue Gnostics, Vienna, Austria).

### 2.13. Conventional Flow Cytometry

#### 2.13.1. Nanoparticle Internalization

Cells were treated with either free Dox or Dox-loaded NPs for various time intervals and detached using Trypsin- 0.05% EDTA solution (Gibco, Waltham, MA, USA). After PBS washing, cells were analyzed using a BD FACSVerse™ Flow Cytometer (BD Biosciences, San Jose, CA, USA). The signal emitted by Dox was detected on the PerCP-Cy5.5 channel.

#### 2.13.2. Cell Cycle

Cells treated for 48 h with either free Dox or Dox-loaded NPs were harvested using Trypsin- 0.05% EDTA solution. The cells in suspension were stained with 10 µg/mL Hoechst at 37 °C and analyzed using a BD FACSAria™ III Cell Sorter (BD Biosciences) on the DAPI channel to quantify the percentage of cells in each cell cycle phase. The signal emitted by Dox was detected on the PerCP-Cy5.5 channel, while Hoechst was detected on the DAPI channel.

#### 2.13.3. Apoptosis

For apoptosis investigation, culture supernatants and washes were collected to retain any cells lifting from the cultures due to apoptosis and added to the cells harvested by trypsinization. After centrifugation, cells were resuspended in Annexin V Binding Buffer (10 mM HEPES, 140 mM NaCl, 2.5 mM CaCl_2_- pH = 7.4) before staining for 15 min with Annexin V- FITC reagent (BD Biosciences) for early apoptotic cell detection. After this interval, cells were diluted 1:4 with buffer and acquired immediately on the BD FACSVerse™ Flow Cytometer (BD Biosciences). The signal emitted by Dox was detected on the PerCP-Cy5.5 channel, while Annexin V^+^ cells were detected on the FITC channel.

#### 2.13.4. Phosphoflow for pERK Expression Analysis

Cells treated with free Dox or Dox-loaded NPs were fixed using 16% paraformaldehyde solution added directly to cell culture medium to reach a final concentration of 1.5% fixation reagent. Cells were detached by scraping and incubated for 10 min at RT. After centrifugation, cells were permeabilized using ice-cold methanol and stored at −80 °C until staining. Cells were washed twice with 0.5% bovine serum albumin (BSA) solution prepared in PBS and incubated with primary antibody anti-phosphoERK (dilution 1:100) for 30 min at RT. After a washing step to remove the unbound antibodies, the samples were stained with secondary antibodies conjugated with AlexaFluor488 (dilution 1:3000). The signal emitted by Dox was detected on the PerCP-Cy5.5 channel, while the signal for phosho-ERK was detected on the FITC channel.

All cytometry data were analyzed using the Cytobank cloud-based platform (https://community.cytobank.org), accessed on 4 December 2019.

### 2.14. Imaging Flow Cytometry

In order to analyze the intracellular localization of fluorescent signals for pERK expression and Dox, we used the Amnis FlowSight imaging flow cytometer and the corresponding acquisition and analysis software packages (IDEAS 6.3 and INSPIRE 200.0.324.0, respectively) (Luminex, accela, Prague, Czech Republic). Cells were treated as in 2.13.4 and manipulated according to manufacturer recommendations. Representative captured cell images are presented together with scattergrams and histograms depicting Dox and pERK signal intensities. Image compensation was conducted on single-stained control samples and applied to all files prior to cytometry analysis.

## 3. Results and Discussion

### 3.1. Physico-Chemical Characterization of Magnetic Nanoparticles

**Figure 1:**
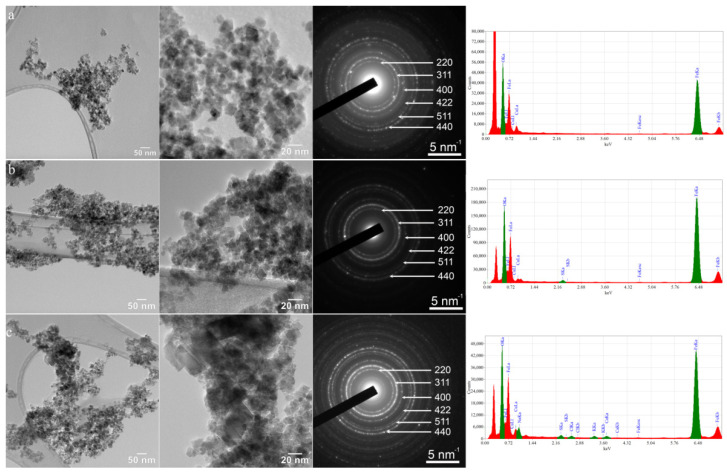
CTEM images, SAED patterns and EDXS spectra for: (**a**) Fe_3_O_4_, (**b**) Fe_3_O_4_-L-Cys and (**c**) Fe_3_O_4_-L-Cys-Dox NPs.

Figure 1 shows CTEM images, SAED patterns and EDXS spectra of Fe_3_O_4_ NPs, Fe_3_O_4_-L-Cys NPs and Fe_3_O_4_-L-Cys-Dox NPs, respectively. CTEM images of all samples (Figure 1, first rows) reveal that NPs have two types of morphologies: spherical shape (for the finest ones) and faceted (cubes or rhombohedral). The size distribution of NPs estimated from CTEM images is quite wide, e.g., between 3 and 20 nm for Fe_3_O_4_ and Fe_3_O_4_-L-Cys NPs, and between 3 and 34 nm for Fe_3_O_4_-L-Cys-Dox NPs.

The SAED patterns displayed in Figure 1 prove that NPs are well crystallized in the magnetite structure (*Fd-3m* space group and lattice parameters a = b = c= 8.375(2) Å). EDS spectra show that all samples contain Fe and O. The spectra for NPs functionalized with L-Cys (Figure 1b) reveal the presence of S, while the one for Fe_3_O_4_-L-Cys-Dox demonstrates the existence of S, Cl, K and Ca (Figure 1c). 

**Figure 2:**
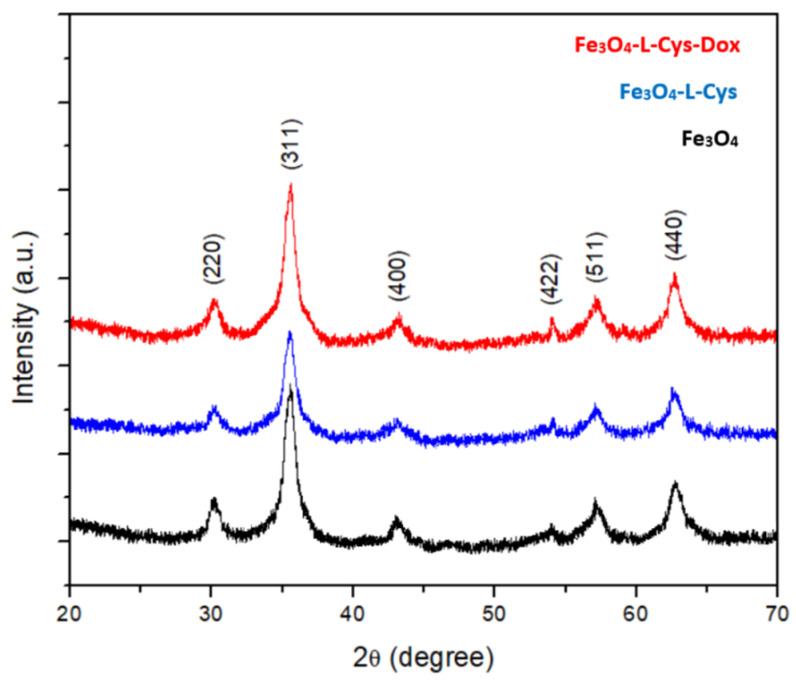
XRD patterns of Fe_3_O_4_, Fe_3_O_4_-L-Cys and Fe_3_O_4_-L-Cys-Dox NPs.

The XRD patterns of synthesized Fe_3_O_4_, Fe_3_O_4_-L-Cys and Fe_3_O_4_-L-Cys-Dox nanoparticles are shown in Figure 2. It can be observed that all the diffraction peaks are well indexed to the planes of (220/2θ = 30°338′), (311/2θ = 35°737′), (400/2θ = 43°438′), (422/2θ = 53°901′), (511/2θ = 57°464′) and (440/2θ = 63°112′) of the pure spinel cubic structure of magnetite with *Fd-3m* space group (ICDD 04-013-9811). In addition, the XRD results show that the crystallographic phase and nature of the Fe_3_O_4_ nanoparticles was not changed after the functionalization with L-cysteine and doxorubicin [39]. The average size of crystallites calculated using the Williamson–Hall method [40] was 4.5 nm, 4.9 nm and 5.4 nm for Fe_3_O_4_, Fe_3_O_4_-L-Cys and Fe_3_O_4_-L-Cys-Dox, respectively. The XRD results are in good agreement with the SAED measurements and confirm that the only crystallographic phase belonged to magnetite structure.

**Figure 3:**
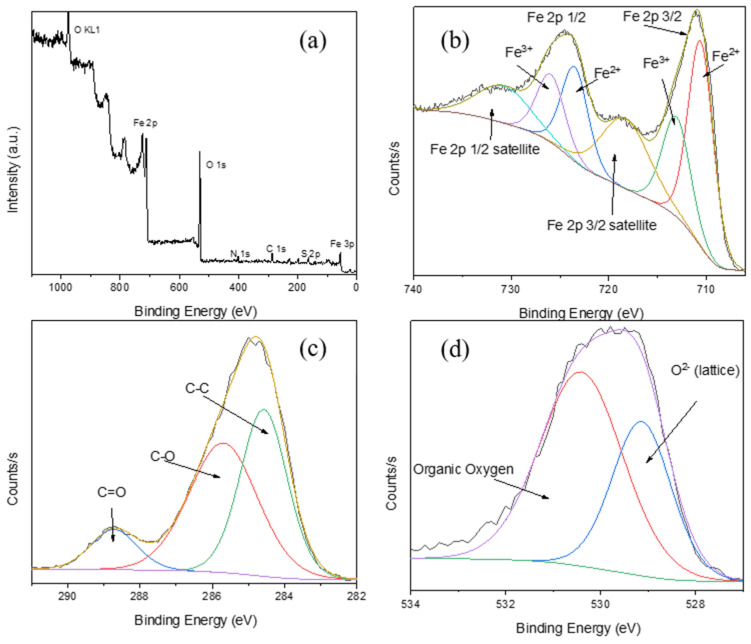
XPS analysis of Fe_3_O_4_ functionalized with L-Cys. (**a**) Survey spectrum and high-resolution spectra of (**b**) Fe 2p, (**c**) C 1 s and (**d**) O 1 s.

The XPS results of Fe_3_O_4_-L-Cys can be visualized in Figure 3. The survey spectrum (Figure 3a) of the L-Cys functionalized NPs shows the presence of the specific elements: O, C, S, Fe and N. In Figure 3b, the high-resolution spectrum of Fe 2p is represented. The binding energy at 710.4 eV corresponds to Fe^2+^ (Fe 2p 3/2) [41,42], whereas the peak at 711.4 eV is assigned to Fe^3+^ (Fe 2p 3/2) [42,43] from the magnetite structure. The peaks at ~286 and ~288.5 eV from the C 1 s and O 1 s [42,44] spectra correspond to C-O and C = O bonds, and confirm the presence of L-Cys on the surface of the NPs. The XPS results prove that the surface of the NPs is functionalized with L-Cys, used further to bind Dox.

### 3.2. Magnetic Properties of the Nanoparticles

**Figure 4:**
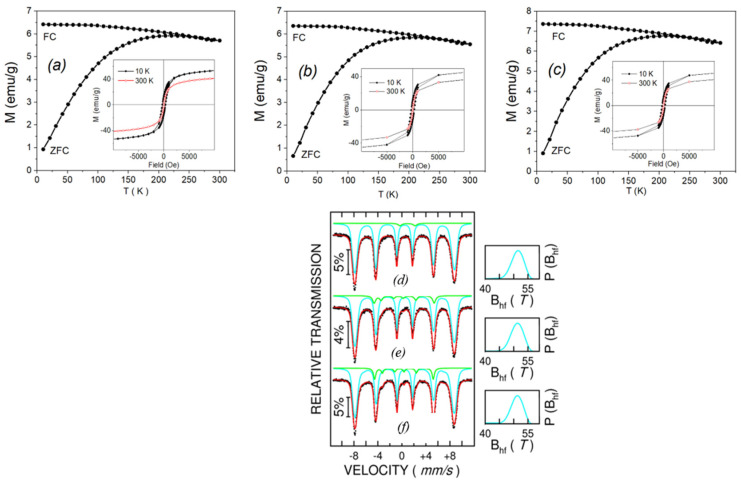
ZFC-FC curves in 100 Oe and hysteresis loops at 10 K (black line) and 300 K (red dashed line) for (**a**) Fe_3_O_4,_ (**b**) Fe_3_O_4_-L-Cys and (**c**) Fe_3_O_4_-L-Cys-Dox NPs. ^57^Fe Mössbauer spectra collected at 6 K on (**d**) Fe_3_O_4_, (**e**) Fe_3_O_4_-L-Cys and (**f**) Fe_3_O_4_-L-Cys-Dox NPs.

The ZFC–FC curves for all three types of samples, measured in 100 Oe, are shown in Figure 4a–c, whereas the hysteresis loops collected at 10 K and 300 K are shown in the corresponding insets. According to these data, the coercive fields of all samples decrease from about 300 Oe at 10 K to about 15 Oe at 300 K, in agreement with an enhanced magnetic relaxation at increased temperature, which, however, is still not complete either at RT. The divergence between the ZFC and FC curves at low temperature and the observed maximum of the ZFC curve point to a magnetic relaxation regime specific to fine NPs [45]. The maximum of the ZFC curve was reached at about 200 K in all cases, which might be considered as an average blocking temperature T_B_. Due to the very flat configuration of the maximum, as well as to the smooth divergence of the two curves, a broad size distribution of NPs can be assumed, which is in accordance with the TEM results. In fact, they? have a broad distribution of the magnetic anisotropy energy of NPs, KV, where K is the anisotropy constant and V the volume of NPs. A broad distribution of anisotropy energies means not only a broad size distribution but also a distribution of the anisotropy constants, most probably connected with the various shapes and sizes of the considered NPs. Noteworthy, by increasing the temperature a long way above the blocking temperature, the ZFC and FC magnetization curves do not decrease at zero but they still keep a significant value of less than 10% lower than the maximum magnetization of the FC curve. This behavior suggests that even at 300 K, only a fraction of the NPs is in the superparamagnetic state, the rest of them being below their own blocking temperature. 

From the magnetic hysteresis loops at 10 K, a saturation magnetization in the interval from 52 to 60 emu/g can be deduced for all samples, values which are some 60% from the specific value of a well-formed magnetite [46]. This is consistent with either an unusual phase composition in the sample or with the formation of a defect magnetite of lower magnetic moment per formula unit (f.u.). In order to explain this aspect, low-temperature Mössbauer spectra were collected. The spectra collected at 6 K on Fe_3_O_4_, Fe_3_O_4_-L-Cys and Fe_3_O_4_-L-Cys-Dox NPs are presented in Figure 4 d, f and g, respectively. 

It can be observed that the 6 K spectrum of simple Fe_3_O_4_ NPs consists of a broad sextet (98% relative spectral area) and a negligible central doublet (only 2% relative spectral area), the last one being assigned by its hyperfine parameters, isomer shift (IS) of 1.00 mm/s, and quadrupole splitting (QS) of 2.4 mm/s, to paramagnetic Fe^2+^ positions, most probable at the particle surface. Concerning the broad magnetic sextet, this was fitted via a hyperfine magnetic field distribution (presented on the right-hand side of the spectrum), characterized by a most probable hyperfine magnetic field of 51.2(2) T and an average IS of 0.45(2) mm/s. These values give support for the assignment of this spectral component to Fe ions in a defect magnetite (close to maghemite) [47]. However, even in this case, the 40% lower value of the magnetization as compared to a well-formed magnetite can be explained only by the formation of a magnetic dead layer with randomly oriented spins of mostly Fe^3+^ ions at the nanoparticle surface.

The 6 K Mössbauer spectrum of Fe_3_O_4_-L-Cys NPs was fitted, beside the broad external sextet (with most probable hyperfine magnetic field of 51.0(2) T and average IS = 0.45(2) mm/s), by an additional inner sextet with hyperfine parameters (hyperfine magnetic field of 31.0(2) T, QS = 0.9 mm/s and IS = −0.06 mm/s) specific to Fe^2+^ ions in an intermediate spin state. Its relative spectral area is of 11(1)%. This additional component may be assigned to those most active Fe positions interacting with active centers of the L-Cys molecules, leading to the formation of Fe^2+^ in very distorted atomic positions. However, such positions can be found only at the NPs’ surface, namely in the magnetic dead layer, and, therefore, cannot significantly modify the magnetic moment of the nanoparticle (and its average magnetization). Finally, the 6 K Mössbauer spectrum of Fe_3_O_4_-L-Cys-Dox NPs is very similar to that of Fe_3_O_4_-L-Cys NPs (almost the same relative spectral area of the additional inner sextet), giving support for the interaction of the Dox molecule with the L-Cys molecules already bound to the Fe ions in the Fe_3_O_4_NPs. 

**Figure 5:**
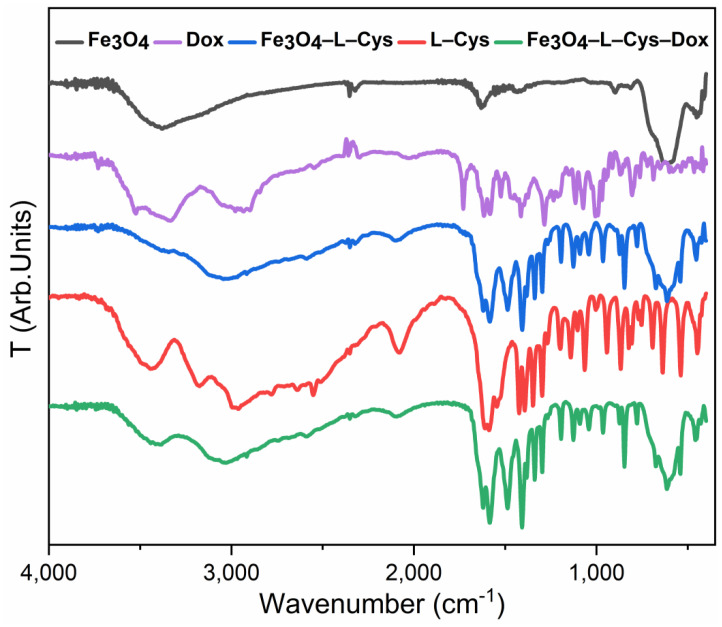
FT-IR spectrum for Fe_3_O_4_, Fe_3_O_4_-L-Cys, Fe_3_O_4_-L-Cys-Dox, Dox and L-Cys.

FT-IR spectroscopy was performed to identify the functional groups of Dox and L-Cys (used as a binder and capping agent in the synthesis of magnetic NPs) (Figure 5). The presence of Fe-O stretching vibration of tetrahedral sites of spinel structure, confirmed by the two strong absorption bands around 573 and 618 cm^−1^, proves the magnetic NPs’ formation [48]. In the case of L-Cys, the characteristic $\upsilon_{as}$ asymmetric (~1591 cm^−^^1^) and $\upsilon_{s}$ symmetric stretching (~1404 cm^−^^1^) corresponding to the COO- group were identified. Further, the peak at 1583 cm^−^^1^ is attributed to the bending vibration of –NH. A weak band near 2557 cm^−^^1^ assigned to the -SH groups of L-Cys molecules can be observed, and the very broad band of (–NH_2_) and (–CH) stretching is located in the 3000–3500 cm^−^^1^ range. The Fe–O stretching mode of the tetrahedral sites can be identified in the band at 543 cm^−1^, and the band located at 582 cm^−1^ is sharp and symmetric, whereas the Fe–OH vibration band is located at ~677 cm^−1^, which is specific for magnetite structure [29,39,49,50]. 

### 3.3. Validation of Dox Loading and Release

**Figure 6:**
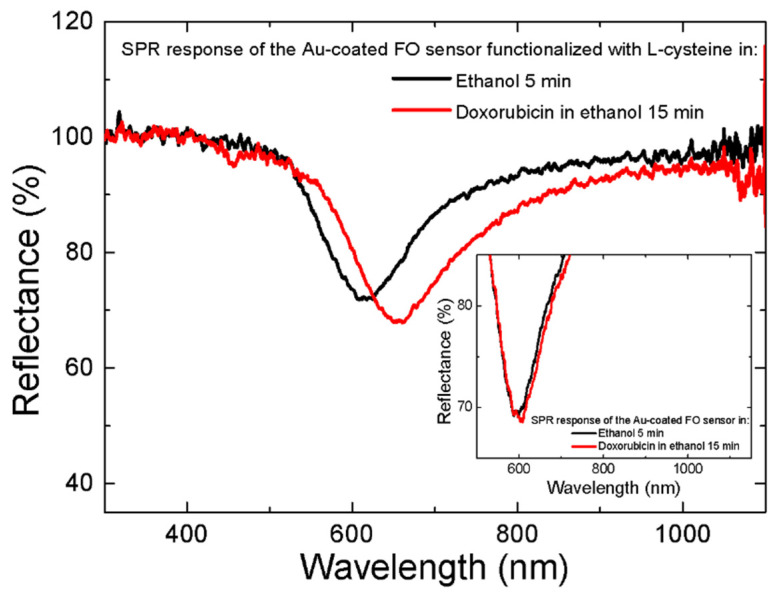
The real-time monitoring of Dox-L-Cys interaction using the FO-SPR system. The SPR spectral dips obtained in ethanol with the sensor functionalized with L-Cys, before (black curve) and after (red curve) Dox binding, respectively. The inset represents a specificity binding test, showing that in the absence of L-Cys on the FO-SPR sensor surface, only a SPR wavelength shift of 4 ± 0.4 nm was recorded for the 0.1 mM Dox solution.

The gold-coated FO-SPR sensors were functionalized with 0.1 M L-Cys and afterwards used to detect 0.1 mM Dox in ethanol. As can be observed in Figure 6, the binding of Dox to L-Cys resulted in an average SPR wavelength shift of 32 ± 0.7 nm. The inset in Figure 6 represents a specificity experiment to verify if Dox (0.1 mM) is binding the gold-coated FO-SPR sensor in the absence of L-Cys. However, a small SPR wavelength shift of 4 ± 0.4 nm could be observed after 15 min, due to the interaction between Dox and the Au-coated sensor surface via the amino (–NH_2_) group.

**Figure 7:**
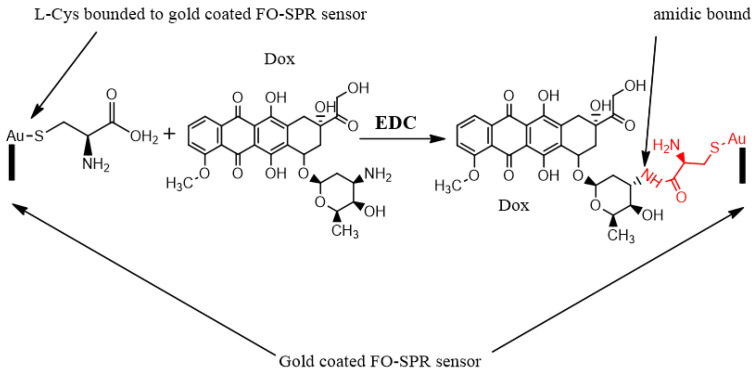
Schematic representation of the chemical interaction between Dox and L-Cys, monitored using the FO-SPR sensor. The L-Cys carboxylic acid is conjugated to doxorubicin amine through amide bonding using EDC (1-ethyl-3-(3-dimethylaminopropyl)carbodiimide) as coupling agent.

The binding mechanism between L-Cys and Dox can be generally explained by the reaction scheme depicted in Figure 7. The crosslinker agent EDC (1-ethyl-3-(3-dimethylaminopropyl)carbodiimide) 0.4 M was used to activate and to couple the carboxyl groups to doxorubicin amine via amide bonding.

**Figure 8:**
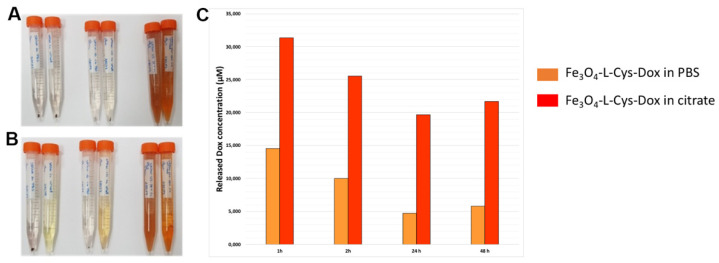
Dox release in PBS buffer (pH = 7.4) and citrate buffer (pH = 3) after (**A**) 1 and (**B**) 24 h of incubation. (**C**) The estimated Dox concentration upon release from the Fe_3_O_4_-L-Cys-Dox NP samples (200 µg/mL) after 1, 2, 24 and 48 h by measuring the fluorescence intensity at 480 nm excitation and 593 nm emission.

Extended evidence supports a decrease in pH within the extracellular tumor micoenvironment as compared to physiological values [51,52]. Moreover, iron-based NPs may encounter acidic organellar pH upon intracellular uptake into cancer cells [53,54]. In order to evaluate the potential variation of NPs’ Dox release response function due to pH, Fe_3_O_4_-L-Cys-Dox NPs were incubated in two buffers with pH values of 7.4 (PBS) and 3 (citrate buffer), respectively. The samples were incubated for up to 48 h, and the release of Dox was determined using UV-Vis spectrometry after 1, 2, 24 and 48 h. Further, the amount of Dox released cumulatively from NP solutions (200 µg/mL concentration) was determined. As it can be seen from Figure 8A and B, after 1 and 24 h of incubation, the Fe_3_O_4_-L-Cys-Dox samples acquired an orange color. The spectrofluorimetric quantification indicates an enhanced release of Dox in the acidic environment for all tested intervals (Figure 8C). One can see that the concentration of Dox released in the first hours of incubation was of 10–15 µM/h (from a solution of 200 µg/mL NPs) for Fe_3_O_4_-L-Cys-Dox, which is expected to induce an increased cytotoxic effect on melanoma cells. The effect should be even more intense in the acidic tumor environment, which would allow a much lower dose of the drug-loaded NPs to be applied. Further work will be dedicated to assess the release capacity of our formulation in vivo in animal models.

### 3.4. Dox Uptake by Fe_3_O_4_-L-Cys-Dox-Treated Melanoma Cells

**Figure 9:**
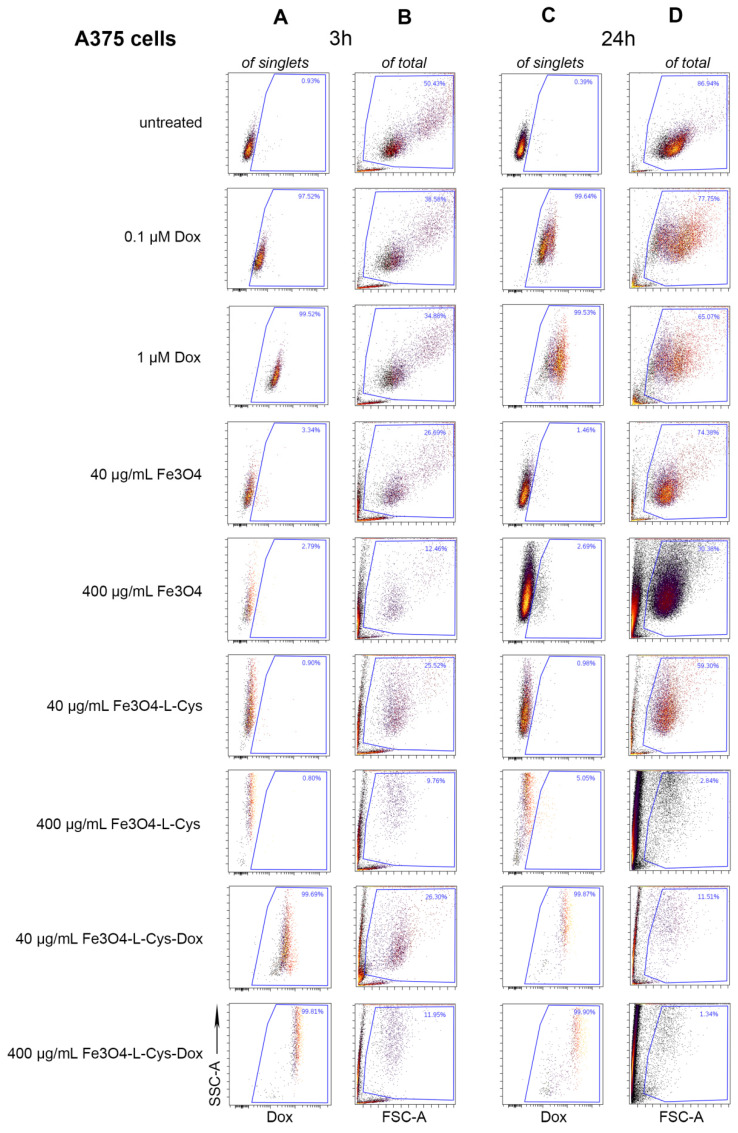
Flow cytometry quantification of Dox uptake by melanoma cells at 3 (**A**,**B**) and 24 h (**C**,**D**) after treatment with drug-loaded NPs versus free drug. Detection of Dox in A375 human melanoma cells (**A**,**C**) was measured on the Per-CP-Cy5.5 channel. Light scattering modifications of A375 cells (**B**,**D**) upon treatment were assessed using FSC-A/SSC-A measurements. FSC = forward-scattered light; SSC = side-scattered light.

The effect of Fe_3_O_4_-L-Cys-Dox NP treatments was investigated on two model melanoma cell lines: human A375 cells and mouse B16F10 cells. First, we determined the drug uptake and retention capacity of melanoma cells versus normal dermal fibroblasts after 3 and 24 h treatment with either Fe_3_O_4_-L-Cys-Dox or controls: Fe_3_O_4_-L-Cys, Fe_3_O_4_ or free Dox. Flow cytometry experiments showed that Dox was taken up by 99–100% of melanoma cells as early as 3 h post-treatment with 40 or 400 μg/mL Fe_3_O_4_-L-Cys-Dox NPs (Figure 9A and Figure S1A) and retained up to 24 h (Figure 9C and Figure S1C) with mean intensity (MFI) values equal to or higher than those obtained using 1 μM free Dox. This was accompanied by a progressive increase in cell granularity (Figure 9B,D and Figure S1B,D—SSC-A), starting with the earliest timepoint, most probably attributable to Fe_3_O_4_-L-Cys NP uptake. As a comparison, free Dox produced a measurable increase in cell granularity at 24 h, and this was more pronounced in A375 cells than in B16F10 cells (Figure 9D versus Figure S1D), and lower than in activated NP-treated cells. When measuring drug uptake into normal dermal fibroblasts, we observed Dox uptake of both free and Fe_3_O_4_-L-Cys-linked drug at 3 h (Figure S2A), similar to the melanoma cells’ behavior (Figure 9A and Figure S1A). However, by 24 h, all fibroblasts retained free Dox, while only 31–45% of cells retained Dox delivered by NPs (Figure S2C). Activated NP uptake (with or without drug) is associated with an increase in granularity (Figure S2B,D), similar to melanoma cells, with Dox producing effects only for the higher concentration at 24 h. These results support the hypothesis that Dox is pumped out by stromal cells in the extracellular space, while melanoma cells retain the drug upon delivery by Fe_3_O_4_-L-Cys NPs, consistent with a potentially decreased cytotoxic effect for non-cancerous cells [55].

**Figure 10:**
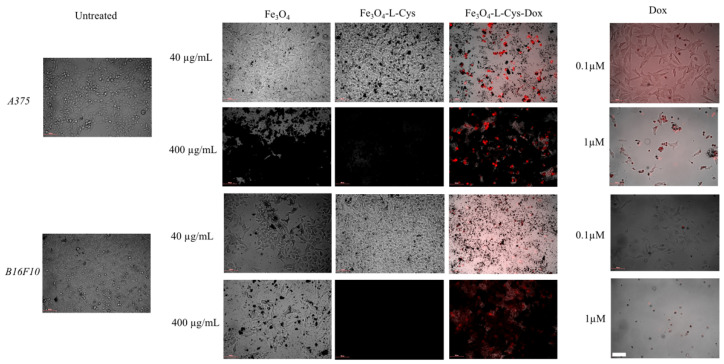
Dox internalization upon treatment with drug-loaded NPs versus free drug. Brightfield superimposed with immunofluorescence images, showing the presence of Dox in cells 48 h after treatment. Scale bar = 100 μm.

Next, we evaluated the morphological changes in cells treated with Fe_3_O_4_-L-Cys-Dox NPs as compared with free Dox or unloaded controls. We found that Fe_3_O_4_-L-Cys-Dox NPs accumulated in the melanoma cells up to 48 h, with a higher signal in A375 cells as compared to B16F10 (Figure 10); a decreased cell density is observed specifically in Fe_3_O_4_-L-Cys-Dox, as well as free-Dox-treated cells, which indicates a cytotoxic effect due to mitosis inhibition at the concentration of 40 µg/mL NPs used, similar to 0.1 µM Dox. Tenfold increase in NP concentration induced a decrease in cell mass even for plain Fe_3_O_4_ treatment. Enlarged cells are visible for 0.1 µM Dox treatment of both melanoma cell lines, and cell death is evident for the 1μM dose.

### 3.5. Cytotoxic and Cytostatic Effects of Fe_3_O_4_-L-Cys-Dox on Treated Melanoma Cells

In order to have the ability to stagnate tumor progression and remove cancer cells from the tumor, an anti-tumor agent must have a cytostatic and/or cytotoxic effect on the cancer cells.

**Figure 11:**
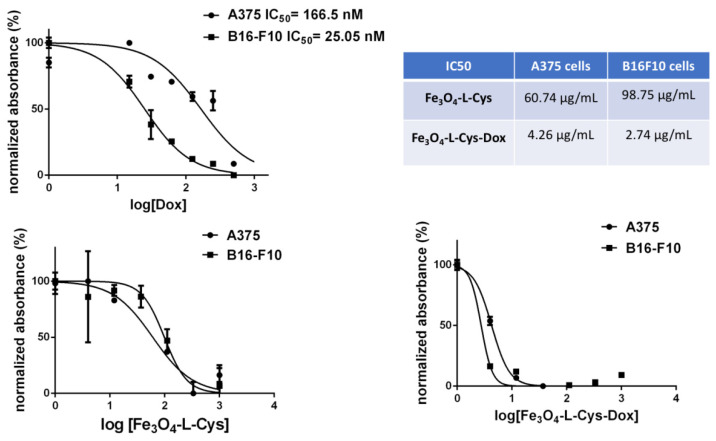
Determination of the half inhibitory concentration (IC50) of Fe_3_O_4_-L-Cys and Fe_3_O_4_-L-Cys-Dox NPs on human A375 and murine B16F10 melanoma cells at 72 h post-treatment, as compared to free Dox.

Next, we tested NPs’ cytotoxic potential by the determination of IC50 values of NP formulations as compared to free drug or plain NPs. The results show that Dox had an IC50 index of ~166 nM on A375 cells and ~25 nM on B16F10 cells (Figure 11). Fe_3_O_4_-L-Cys-Dox nanoparticles showed lower IC50 values than Fe_3_O_4_-L-Cys (Figure 11-table). B16F10 cells were more sensitive than A375 to both free and NPs-delivered Dox treatment, as previously observed using fluorescence microscopy (Figure 10).

**Figure 12:**
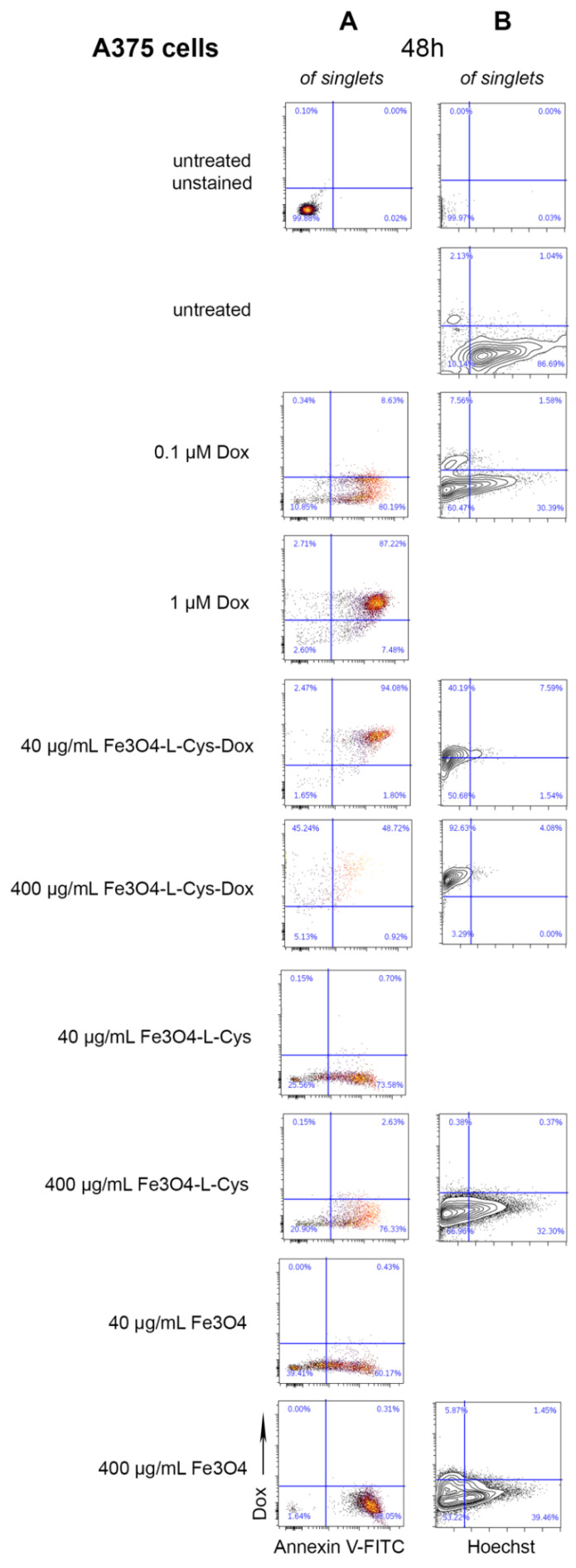
Cytometric evaluation of cytotoxic and cytostatic potential of Fe_3_O_4_, Fe_3_O_4_-L-Cys and Fe_3_O_4_-L-Cys-Dox NPs. Analysis of the percentage of cells in apoptosis (**A**) and the cell cycle profile (**B**) following 48 h treatment with NPs or free Dox in human A375 melanoma cells.

Next, the level of apoptosis of A375 and B16F10 cells was quantified using flow cytometry after treatment for 48 h with Fe_3_O_4_-L-Cys-Dox NPs versus free Dox. Upon 40 µg/mL treatment with Dox-loaded NPs, we observed that 97–99% of melanoma cells retained the drug and were apoptotic at this timepoint (Figure 12A and Figure S3A), while most cells did not survive treatment with 400 µg/mL. Additionally, more than 80% of cells were apoptotic when treated with NPs alone at these concentrations. In comparison, a dose-dependent increase in Dox accumulation was seen in cells treated with free drug (~30% of cells treated with 0.1 µM Dox and ~90% of cells treated with 1µM Dox). More than 90% of cells were apoptotic in these conditions. Hence, treatments with 40 µg/mL Fe_3_O_4_-L-Cys-Dox NPs induce a 95–98% apoptosis of melanoma cells, similar to the effect produced by the free antitumor agent at 0.1–1 µM.

The ability of Fe_3_O_4_-L-Cys-Dox NPs to block melanoma cells during their progression through the cell cycle before division was evaluated in the following experiment by Hoechst staining of cells that remained attached. The data show a blockage of a ~30% A375 cell fraction and of ~90% of B16F10 cells, which became aneuploid after their treatment with 0.1 µM free Dox (Figure 12B and Figure S3B). When 40 or 400 µg/mL Fe_3_O_4_-L-Cys-Dox NPs were applied to A375 cells, only 30% retained their cycling ability, while 65% still remained proliferative when treated with the maximum concentration of Fe_3_O_4_ or Fe_3_O_4_-L-Cys (Figure 12B). In contrast, B16F10 cells were much more sensitive and treatment with these concentrations of NPs totally blocked the cell cycle (Figure S3B).

The results correlate with the data obtained upon investigating the percentage of cells in apoptosis after NP treatment and underline the cytostatic effect of NP formulation with Dox.

### 3.6. Fe_3_O_4_-L-Cys-Dox Induces Changes in ERK Phosphorylation

**Figure 13:**
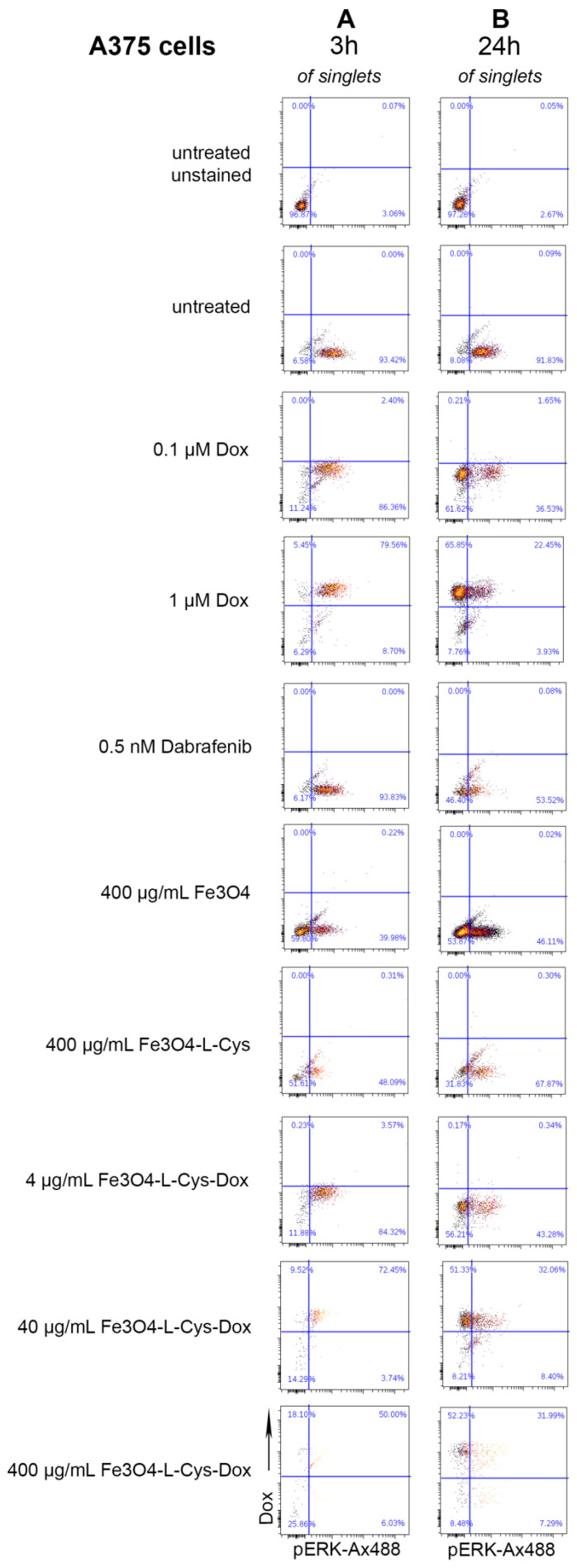
Cytometric evaluation of pERK expression at 3 h (**A**) and 24 h (**B**) after incubation of A375 cells with nanoparticles or free Dox. Phosphorylated ERK was detected on the FITC channel, upon labeling with Alexa Fluor 488-conjugated secondary antibodies, and Dox on the PerCP-Cy5.5 channel.

Due to the fact that Dox blocks DNA replication, the main impact of this drug is on cell proliferation. The main signaling pathway associated with this process in tumor cells, as well as with the apoptosis response to chemotherapeutic drugs, is the MAPK pathway [56]. Therefore, we sought to test the effect of the performed treatments on MAPK activity via ERK phosphorylation (pERK). The A375 cells have the BRAF V600E mutation, which induces a constitutive activation of MAPK, highlighted by the increased expression of pERK (Figure 13A—93% pERK^+^ cells at 3 h). Treatment with 0.1–1 µM free Dox or 4 µg/mL Fe_3_O_4_-L-Cys-Dox did not change significantly ERK activation (~88% pERK^+^). ERK phosphorylation decreased at 76% and 55%, respectively, after treatment with 40 or 400 µg/mL Fe_3_O_4_-L-Cys-Dox. Interestingly, Fe_3_O_4_ or Fe_3_O_4_-L-Cys decreased pERK^+^ cell fraction at 40% and 48%, respectively, when used in excess (400 µg/mL). This indicates that at 3 h, the NPs affect ERK activity in A375 cells and not the drug. By 24 h, the pERK activity decreased at 26–38% of Dox-treated cells, more than in cells treated with 0.5 nM dabrafenib, a specific MAPK inhibitor (Figure 13B, 54% pERK^+^). After treatment with 40 or 400 µg/mL Fe_3_O_4_-L-Cys-Dox, this population represented ~40% of melanoma cells. Fe_3_O_4_ or Fe_3_O_4_-L-Cys also inactivated this pathway by decreasing the pERK^+^ fraction at 46% and 68% of A375 cells, respectively, which shows a dual role of both NPs and Dox, supporting the cell cycle data (Figure 12B).

In comparison, in B16F10 cells, only 51% of cells had active ERK at the 3 h timepoint. Treatment with 1µM Dox slightly increased %pERK^+^ cells to 64%, as compared to 87% in phorbol-myristate-acetate (PMA)-treated cells, which is a known pERK positive regulator (Figure S4A). Dabrafenib largely maintained the basal level of activation (without treatment). Interestingly, pERK^+^ fractions decreased at ~44%, when cells were treated with increasing doses of Fe_3_O_4_-L-Cys-Dox. The highest doses of Fe_3_O_4_ or Fe_3_O_4_-L-Cys also decreased %pERK^+^ that reached only ~28%, which suggests an inhibitory action of the NPs alone on pERK activity at this timepoint. When tested at 24 h, B16F10 cells showed a basal level of 53% pERK^+^ cells. Exposure to 1µM free Dox decreased this fraction to 15%, as compared to 84% for PMA and 34% for Dabrafenib (Figure S4B). Fe_3_O_4_-L-Cys-Dox decreased pERK^+^ fraction only in cells treated for 24 h with 40 or 400 µg/mL NPs (18% and 28%, respectively). This effect is attributable to the loaded Dox, as NPs alone maintained 40% of pERK^+^ fraction.

**Figure 14:**
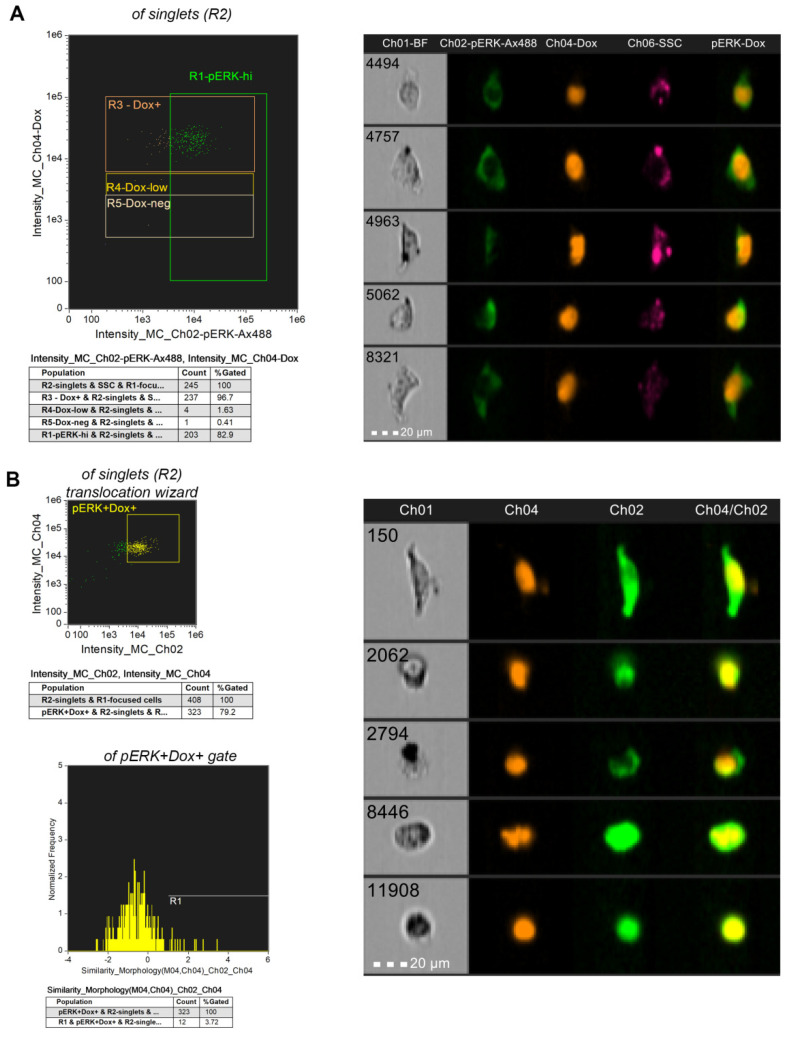
Imaging flow cytometry analysis of pERK expression at 3 h after incubation of A375 cells with 4 µg/mL Fe_3_O_4_-L-Cys-Dox nanoparticles. Phosphorylated ERK was detected on channel 02, and Dox on channel 04. Merged Ch02/Ch04 fluorescence, single-channel brightfield (Ch01) and side scatter (SSC, Ch06) images are provided. Dot plots as well as representative images of single cells are shown for each analysis. (**A**) R1 gate marks cells with high ERK activation, irrespective of Dox uptake level. Events from this gate are exemplified in the corresponding images. (**B**) Gated cells represent pERK^+^ cells that have taken up the drug. Cells expressing pERK in the nucleus were analyzed using the translocation wizard (defined as R1). R1 events are exemplified in the last two rows of images in each data set.

Next, we used imaging flow cytometry to evaluate ERK phosphorylation in A375 and B16F10 cells after treatment for 3 h with 4µg/mL Fe_3_O_4_-L-Cys-Dox NPs. Selected images of pERK-hi gates are provided for detailed examination of Dox and pERK signal, and their respective intracellular localizations (Figure 14 and Figure S5). Moreover, we were able to reveal NPs clusters in the melanoma cells which correlate with increased SSC signal, as in conventional flow cytometry (Ch-01 BF vs. Ch06-SSC). In Figure 14A and Figure S5A, a higher % of pERK-hi cells are visible in Dox^+^ A375 than in Dox^+^ B16F10 cells, consistent with the conventional flow cytometry results (Figure 13A and Figure S4A). For these specific populations that had taken up the drug and have the highest pERK signal, five representative images were selected. These show that delivered Dox is accumulated in the nucleus, while pERK signal is in the cell cytoplasm for the majority of cells. Additionally, pERK signal is higher in A375 cells than in B16F10. Using the translocation wizard, we were able to identify the cells that have translocated pERK in the nucleus (Figure 14B and Figure S5B). These represent 3.72% of analyzed cells in A375 and 12.8% of B16F10 cells from the pERK^+^Dox^+^ cell populations. Cells on the last two image rows depict these events in Ch02. This difference might be explained by the oversaturated ERK phosphorylation in A375 cells as compared to B16F10 cells. This set of results brings more evidence that Fe_3_O_4_-L-Cys-Dox NPs are able to deliver Dox inside melanoma cells and it accumulates in the nucleus, where it impacts cell function.

## 4. Conclusions

Here, we propose L-Cys as a new type of binder for drug loading onto Fe_3_O_4_ NPs. The NP formulation was thoroughly investigated and fully characterized with respect to morpho-structural, local electronic and magnetic properties. The interaction of L-Cys with the NPs’ surface was proven using XPS, while the binding of Dox to L-Cys was demonstrated using FO-SPR monitoring.

The effect of Fe_3_O_4_-L-Cys-Dox NP treatments was investigated on two model melanoma cell lines: human A375 cells and mouse B16F10 cells. The flow cytometry experiments showed a rapid uptake of Fe_3_O_4_-L-Cys-Dox NPs in melanoma cells within 3 h of treatment, which produced apoptotic effects detectable at 48 h post-exposure, leading to cell death in almost all melanoma cells. Interestingly, 60–80% of dermal fibroblasts took up the NP-delivered drug in the first hours after treatment, but only 30–45% retained it by 24 h; melanoma cells, however, retained Dox, which further led to cancer cell apoptosis. Half inhibitory concentrations (IC50 values) were determined around 2–4 µg/mL for Fe_3_O_4_-L-Cys-Dox NPs for the tested cell lines.

Apoptosis and cell cycle analysis demonstrated the cytotoxic and cytostatic effect of Fe_3_O_4_-L-Cys NP formulation containing Dox. An arrest of melanoma cells in the G0/G1 cell cycle phase correlated with increased apoptosis at 48 h post-treatment.

Imaging flow cytometry confirmed the nuclear accumulation of delivered Dox in both A375 and B16F10 melanoma cell lines, while revealing cytoplasmic accumulation of nanoparticle clusters. Both conventional and imaging flow cytometry showed an increased level of MAPK pathway activation in A375 as opposed to B16F10, consistent with the mutational status of BRAF (BRAFV600E in A375 vs. BRAFwt in B16F10). Our results demonstrate the capacity of Fe_3_O_4_-L-Cys-Dox NPs to modulate cell function by interfering with key signaling events—a dose-dependent decrease in ERK phosphorylation by exposure to drug-loaded NPs as compared to untreated cells was observed in both cell lines at 3 and 24 h post-treatment. Mechanistically, after treatment for 3 h with 4 µg/mL Fe_3_O_4_-L-Cys-Dox, out of the pERK^+^ cell fraction, only 3–12% showed nuclear translocation of the phosphorylated signaling protein in both cell lines tested.

Subsequent experiments will be performed in animal models *in vivo* to determine the specificity of cancer cell targeting, the ability to prevent tumor progression, and the capacity to extend life span.

## Data Availability

Not applicable.

## Supplementary Materials

The following supporting information can be downloaded at: https://www.mdpi.com/article/10.3390/nano13040621/s1, Figure S1: Flow cytometry quantification of doxorubicin (Dox) uptake by human CCD dermal fibroblasts at 3 h (A,B) and 24 h (C,D) after treatment with drug-loaded nanoparticles versus free drug. Detection of Dox (A,C) was measured on the Per-CP-Cy5.5 channel. Light scattering modifications upon treatment were assessed using FSC-A/SSC-A measurements. FSC = forward-scattered light; SSC—side-scattered light. Figure S2: Flow cytometry quantification of Doxorubicine (Dox) uptake by human CCD dermal fibroblasts at 3 h (A,B) and 24 h (C,D) after treatment with drug loaded nanoparticles versus free drug. Detection of Dox (A,C) was measured into the Per-CP-Cy5.5 channel. Light scattering modifications upon treatment were assessed by FSC-A/SSC-A measurements. FSC = forward scattered light; SSC—side scattered light. Figure S3: Cytometric evaluation of cytotoxic and cytostatic potential of Fe_3_O_4_, Fe_3_O_4_-L-Cys, and Fe_3_O_4_-L-Cys-Dox NPs. Analysis of the percentage of cells in apoptosis (A) and the cell cycle profile (B) following 48 h treatment with NPs or free Dox in mouse B16F10 melanoma cells. , Figure S4: Cytometric evaluation of pERK expression at 3 h (A) and 24 h (B) after incubation of B16F10 cells with nanoparticles or free Dox. Phosphorylated ERK was detected on the FITC channel, upon labeling with Alexa Fluor 488-conjugated secondary antibodies, and Dox on the PerCP-Cy5.5 channel. Figure S5: Imaging flow cytometry analysis of pERK expression at 3 h after incubation of B16F10 cells with 4 µg/mL Fe_3_O_4_-L-Cys-Dox nanoparticles. Phosphorylated ERK was detected on channel 02, and Dox on channel 04. Merged Ch02/Ch04 fluorescence, as well as single channel brightfield (Ch01) and side scatter (SSC, Ch06) images are provided. Dot plots as well as representative images of single cells are shown for each analysis. (A) R1 gate marks cells with high ERK activation, irrespective of Dox uptake level. Events from this gate are exemplified in the corresponding images. (B) Gated cells represent pERK^+^ cells that have taken up the drug. Cells expressing pERK in the nucleus were analyzed using the translocation wizard (defined as R1). R1 events are exemplified in the two last rows of images in each data set.

## References

[1] Dasari S., Yedjou C.G., Brodell R.T., Cruse A.R., Tchounwou P.B. (2020). Therapeutic strategies and potential implications of silver nanoparticles in the management of skin cancer. Nanotechnol. Rev..

[2] Marks R. (2000). Epidemiology of melanoma. Clin. Exp. Dermatol..

[3] Leiter U., Eigentler T., Garbe C., Reichrath J. (2014). Epidemiology of Skin Cancer. Sunlight, Vitamin D and Skin Cancer.

[4] Dianzani C., Zara G.P., Maina G., Pettazzoni P., Pizzimenti S., Rossi F., Gigliotti C.L., Ciamporcero E.S., Daga M., Barrera G. (2014). Drug Delivery Nanoparticles in Skin Cancers. Biomed Res. Int..

[5] Kievit F.M., Wang F.Y., Fang C., Mok H., Wang K., Silber J.R., Ellenbogen R.G., Zhang M. (2011). Doxorubicin loaded iron oxide nanoparticles overcome multidrug resistance in cancer in vitro. J. Control. Release.

[6] Yeganeh F.E., Yeganeh A.E., Far B.F., Mansouri A., Sibuh B.Z., Krishnan S., Pandit S., Alsanie W.F., Thakur V.K., Gupta P.K. (2022). Synthesis and Characterization of Tetracycline Loaded Methionine-Coated NiFe_2_O_4_ Nanoparticles for Anticancer and Antibacterial Applications. Nanomaterials.

[7] Edis Z., Wang J., Waqas M.K., Ijaz M., Ijaz M. (2021). Nanocarriers-Mediated Drug Delivery Systems for Anticancer Agents: An Overview and Perspectives. Int. J. Nanomed..

[8] Din F.U., Aman W., Ullah I., Qureshi O.S., Mustapha O., Shafique S., Zeb A. (2017). Effective use of nanocarriers as drug delivery systems for the treatment of selected tumors. Int. J. Nanomed..

[9] Lei W., Yang C., Wu Y., Ru G., He X., Tong X., Wang S. (2022). Nanocarriers surface engineered with cell membranes for cancer targeted chemotherapy. J. Nanobiotechnol..

[10] Gour V., Agrawal P., Pandey V., Kanwar I.L., Haider T., Tiwari R., Soni V., Yadav A.K., Gupta U., Sharma R. (2021). Chapter 10-Nanoparticles and skin cancer. Nano Drug Delivery Strategies for the Treatment of Cancers.

[11] Jain S.K., Haider T., Kumar A., Jain A. (2016). Lectin-Conjugated Clarithromycin and Acetohydroxamic Acid-Loaded PLGA Nanoparticles: A Novel Approach for Effective Treatment of H. pylori. AAPS PharmSciTech.

[12] Lee W.G., Kim Y.-G., Chung B.G., Demirci U., Khademhosseini A. (2010). Nano/Microfluidics for diagnosis of infectious diseases in developing countries. Adv. Drug Deliv. Rev..

[13] Raj S., Jose S., Sumod U.S., Sabitha M. (2012). Nanotechnology in cosmetics: Opportunities and challenges. J. Pharm. Bioallied Sci..

[14] Soni V., Kohli D.V., Jain S.K. (2005). Transferrin coupled liposomes as drug delivery carriers for brain targeting of 5-florouracil. J. Drug Target..

[15] Zhao Q.-H., Zhang Y., Liu Y., Wang H.-L., Shen Y.-Y., Yang W.-J., Wen L.-P. (2010). Anticancer effect of realgar nanoparticles on mouse melanoma skin cancer in vivo via transdermal drug delivery. Med. Oncol..

[16] Dulińska-Litewka J., Łazarczyk A., Hałubiec P., Szafrański O., Karnas K., Karewicz A. (2019). Superparamagnetic Iron Oxide Nanoparticles—Current and Prospective Medical Applications. Materials.

[17] Pandey V., Gajbhiye K.R., Soni V. (2015). Lactoferrin-appended solid lipid nanoparticles of paclitaxel for effective management of bronchogenic carcinoma. Drug Deliv..

[18] Jain A., Jain A., Garg N.K., Tyagi R.K., Singh B., Katare O.P., Webster T.J., Soni V. (2015). Surface engineered polymeric nanocarriers mediate the delivery of transferrin-methotrexate conjugates for an improved understanding of brain cancer. Acta Biomater..

[19] Yang S.-J., Lin F.-H., Tsai K.-C., Wei M.-F., Tsai H.-M., Wong J.-M., Shieh M.-J. (2010). Folic Acid-Conjugated Chitosan Nanoparticles Enhanced Protoporphyrin IX Accumulation in Colorectal Cancer Cells. Bioconjug. Chem..

[20] Ito S., Wakamatsu K. (2003). Quantitative Analysis of Eumelanin and Pheomelanin in Humans, Mice, and Other Animals: A Comparative Review. Pigment Cell Res..

[21] Feng X., Deng C., Gao M., Zhang X. (2018). Facile and easily popularized synthesis of l-cysteine-functionalized magnetic nanoparticles based on one-step functionalization for highly efficient enrichment of glycopeptides. Anal. Bioanal. Chem..

[22] Sadighian S., Rostamizadeh K., Hosseini-Monfared H., Hamidi M. (2014). Doxorubicin-conjugated core–shell magnetite nanoparticles as dual-targeting carriers for anticancer drug delivery. Colloids Surf. B Biointerfaces.

[23] Fratila R.M., Moros M., de la Fuente J.M. (2016). Recent advances in biosensing using magnetic glyconanoparticles. Anal. Bioanal. Chem..

[24] Sun S., Yang G., Wang T., Wang Q., Chen C., Li Z. (2010). Isolation of N-linked glycopeptides by hydrazine-functionalized magnetic particles. Anal. Bioanal. Chem..

[25] Ashour R.M., Abdel-Magied A.F., Abdel-Khalek A.A., Helaly O.S., Ali M.M.N. (2016). Preparation and characterization of magnetic iron oxide nanoparticles functionalized by l-cysteine: Adsorption and desorption behavior for rare earth metal ions. J. Environ. Chem. Eng..

[26] Clemente Plaza N., Reig García-Galbis M., Martínez-Espinosa R.M. (2018). Effects of the Usage of l-Cysteine (l-Cys) on Human Health. Molecules.

[27] Fahey R.C. (1977). Biologically important thiol-disulfide reactions and the role of cyst(e)ine in proteins: An evolutionary perspective. Protein Crosslinking.

[28] Safaei-Ghomi J., Ebrahimi S.M. (2022). Nano-Fe_3_O_4_–Cysteine as a Superior Catalyst for the Synthesis of Indeno[1,2-c]pyrazol-4(1H)-ones. Polycycl. Aromat. Compd..

[29] Bashir A., Pandith A.H., Malik L.A., Qureashi A., Ganaie F.A., Dar G.N. (2021). Magnetically recyclable L-cysteine capped Fe_3_O_4_ nanoadsorbent: A promising pH guided removal of Pb(II), Zn(II) and HCrO_4_- contaminants. J. Environ. Chem. Eng..

[30] Fan L., Deng M., Lin C., Xu C., Liu Y., Shi Z., Wang Y., Xu Z., Li L., He M. (2018). A multifunctional composite Fe_3_O_4_/MOF/L-cysteine for removal, magnetic solid phase extraction and fluorescence sensing of Cd(ii). RSC Adv..

[31] Khalafi-Nezhad A., Nourisefat M., Panahi F. (2015). l-Cysteine functionalized magnetic nanoparticles (LCMNP): A novel magnetically separable organocatalyst for one-pot synthesis of 2-amino-4H-chromene-3-carbonitriles in water. Org. Biomol. Chem..

[32] Liu D., Tan H., Meng L., Jia H., Zhou W., Wu H. (2021). Preparation of Cysteine-Functionalized Fe_3_O_4_ Magnetic Nanoparticles for Determination of Cu^2+^. Chem. Sel..

[33] Mondal L., Mukherjee B., Das K., Bhattacharya S., Dutta D., Chakraborty S., Pal M.M., Gaonkar R.H., Debnath M.C. (2019). CD-340 functionalized doxorubicin-loaded nanoparticle induces apoptosis and reduces tumor volume along with drug-related cardiotoxicity in mice. Int. J. Nanomed..

[34] Antohe I., Jinga L.-I., Antohe V.-A., Socol G. (2021). Sensitive pH Monitoring Using a Polyaniline-Functionalized Fiber Optic—Surface Plasmon Resonance Detector. Sensors.

[35] Antohe I., Iordache I., Antohe V.-A., Socol G. (2021). A polyaniline/platinum coated fiber optic surface plasmon resonance sensor for picomolar detection of 4-nitrophenol. Sci. Rep..

[36] Antohe (Arghir) I., Schouteden K., Goos P., Delport F., Spasic D., Lammertyn J. (2016). Thermal annealing of gold coated fiber optic surfaces for improved plasmonic biosensing. Sens. Actuators B Chem..

[37] Arghir I., Spasic D., Verlinden B.E., Delport F., Lammertyn J. (2015). Improved surface plasmon resonance biosensing using silanized optical fibers. Sens. Actuators B Chem..

[38] Şolomonea B.-G., Jinga L.-I., Antohe V.-A., Socol G., Antohe I. (2022). Cadmium Ions’ Trace-Level Detection Using a Portable Fiber Optic—Surface Plasmon Resonance Sensor. Biosensors.

[39] Bagbi Y., Sarswat A., Mohan D., Pandey A., Solanki P.R. (2017). Lead and Chromium Adsorption from Water using L-Cysteine Functionalized Magnetite (Fe_3_O_4_) Nanoparticles. Sci. Rep..

[40] Williamson G.K., Hall W.H. (1953). X-ray line broadening from filed aluminium and wolfram. Acta Metall..

[41] Oku M., Hirokawa K. (1976). X-ray photoelectron spectroscopy of Co_3_O_4_, Fe_3_O_4_, Mn_3_O_4_, and related compounds. J. Electron Spectros. Relat. Phenom..

[42] Rumble Jr. J. (1992). R.; Bickham, D.M.; Powell, C.J. The NIST X-ray photoelectron spectroscopy database. Surf. Interface Anal..

[43] Allen G.C., Curtis M.T., Hooper A.J., Tucker P.M. (1974). X-ray photoelectron spectroscopy of iron–oxygen systems. J. Chem. Soc. Dalton Trans..

[44] Moulder J.F., Stickle W.F., Sobol W.M., Bomben K.D. (1992). Handbook of X-ray Photoelectron Spectroscopy.

[45] Kuncser V., Miu L. (2014). Size Effects in Nanostructures: Basics and Applications.

[46] Crăciunescu I., Palade P., Iacob N., Ispas G.M., Stanciu A.E., Kuncser V., Turcu R.P. (2021). High-Performance Functionalized Magnetic Nanoparticles with Tailored Sizes and Shapes for Localized Hyperthermia Applications. J. Phys. Chem. C.

[47] Kuncser V., Schinteie G., Sahoo B., Keune W., Bica D., Vekas L., Filoti G. (2007). Magnetic interactions in water based ferrofluids studied by Mössbauer spectroscopy. J. Phys. Condens. Matter.

[48] Pham X.N., Nguyen T.P., Pham T.N., Tran T.T.N., Tran T.V.T. (2016). Synthesis and characterization of chitosan-coated magnetite nanoparticles and their application in curcumin drug delivery. Adv. Nat. Sci. Nanosci. Nanotechnol..

[49] Ma X., Guo Q., Xie Y., Ma H. (2016). Green chemistry for the preparation of l-cysteine functionalized silver nanoflowers. Chem. Phys. Lett..

[50] Kogelheide F., Kartaschew K., Strack M., Baldus S., Metzler-Nolte N., Havenith M., Awakowicz P., Stapelmann K., Lackmann J.-W. (2016). FTIR spectroscopy of cysteine as a ready-to-use method for the investigation of plasma-induced chemical modifications of macromolecules. J. Phys. D Appl. Phys..

[51] Vaupel P., Kallinowski F., Okunieff P. (1989). Blood flow, oxygen and nutrient supply, and metabolic microenvironment of human tumors: A review. Cancer Res..

[52] Wike-Hooley J.L., Haveman J., Reinhold H.S. (1984). The relevance of tumour pH to the treatment of malignant disease. Radiother. Oncol..

[53] Singh N., Jenkins G.J.S., Asadi R., Doak S.H. (2010). Potential toxicity of superparamagnetic iron oxide nanoparticles (SPION). Nano Rev..

[54] Watabe H., Valencia J.C., Yasumoto K.-I., Kushimoto T., Ando H., Muller J., Vieira W.D., Mizoguchi M., Appella E., Hearing V.J. (2004). Regulation of tyrosinase processing and trafficking by organellar pH and by proteasome activity. J. Biol. Chem..

[55] Condello M., Cosentino D., Corinti S., Di Felice G., Multari G., Gallo F.R., Arancia G., Meschini S. (2014). Voacamine modulates the sensitivity to doxorubicin of resistant osteosarcoma and melanoma cells and does not induce toxicity in normal fibroblasts. J. Nat. Prod..

[56] Wellbrock C., Arozarena I. (2016). The Complexity of the ERK/MAP-Kinase Pathway and the Treatment of Melanoma Skin Cancer. Front. Cell Dev. Biol..

