# Supplementary material for: Synthesis and Anti-Melanoma Activity of L-Cysteine-Coated Iron Oxide Nanoparticles Loaded with Doxorubicin
Source: Nanomaterials (Basel). 2023 Feb 4;13(4):621. doi: 10.3390/nano13040621 (PMC9966685; doi:10.3390/nano13040621)
Supplement: Supplementary file 1 [file nanomaterials-13-00621-s001.zip › nanomaterials-2165122-supplementary.pdf]

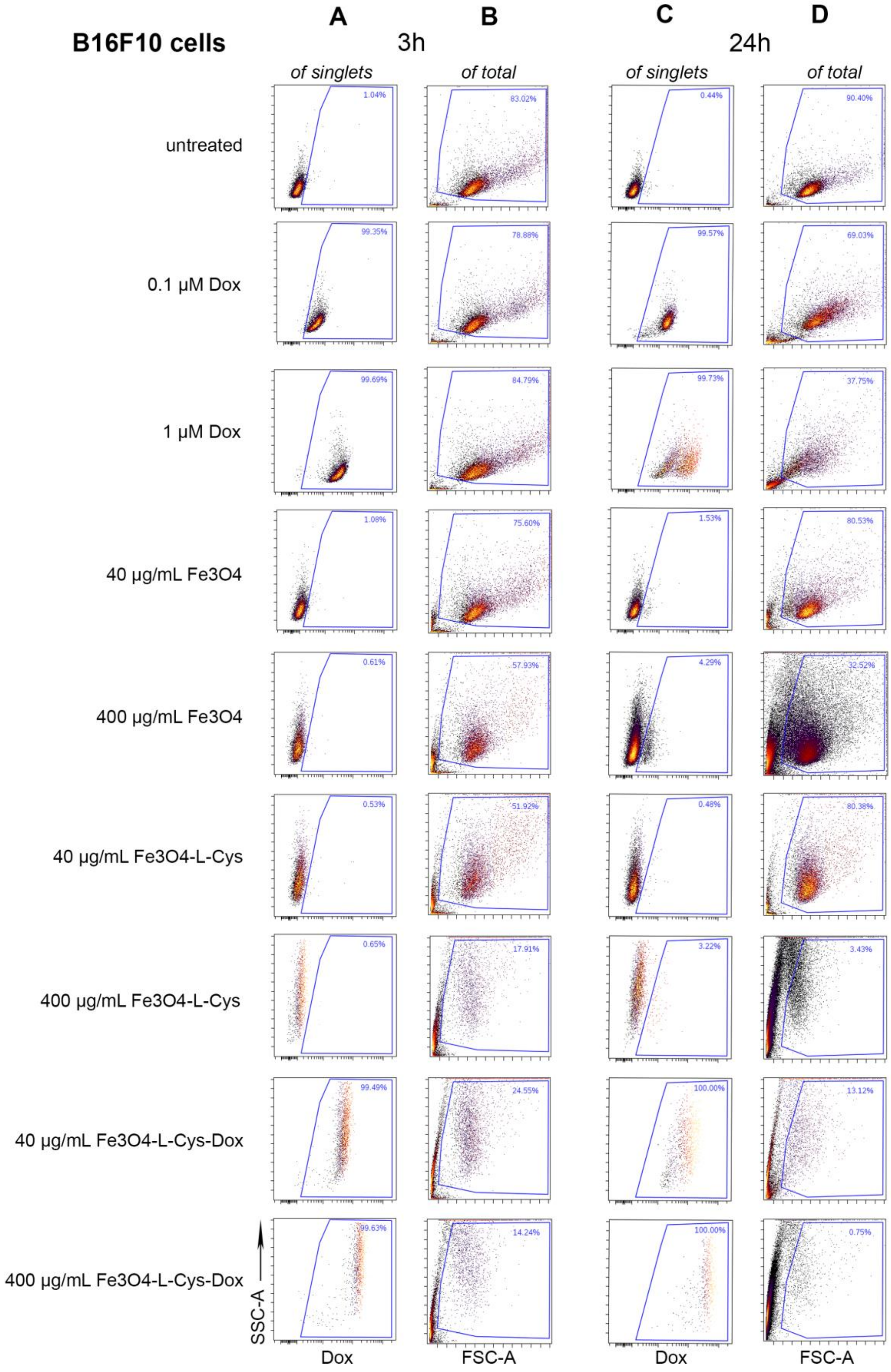

Figure S1. Flow cytometry quantification of Dox uptake by melanoma cells at 3 (A, B) and 24h (C, D) after treatment with drug loaded NPs versus free drug. Detection of Dox in B16F10 mouse melanoma cells (A, C) was measured into the Per-CP-Cy5.5 channel. Light scattering modifications of B16F10 cells (B, D) upon treatment were assessed by FSC-A/SSC-A measurements. FSC = forward scattered light; SSC = side scattered light.

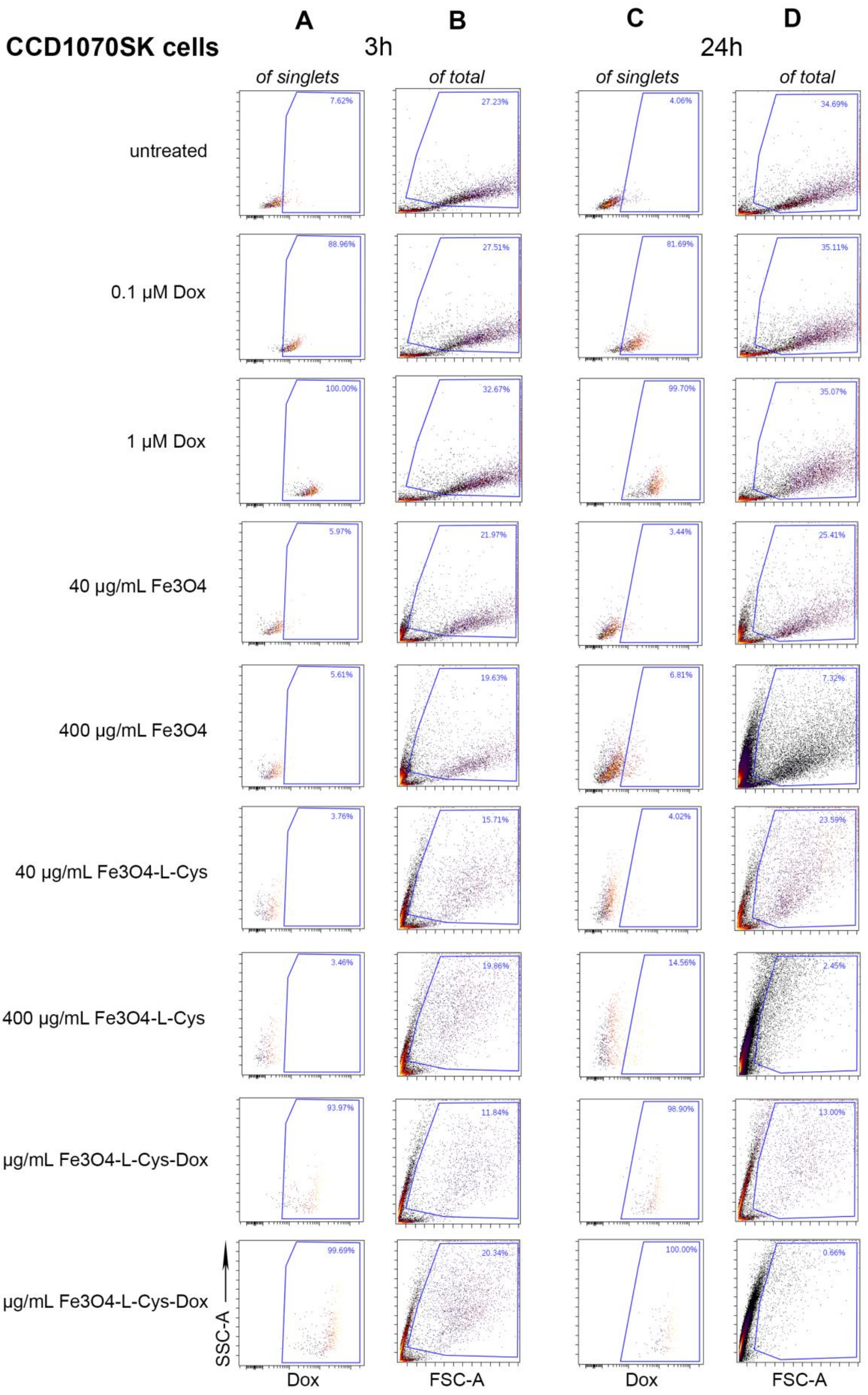

Figure S2: Flow cytometry quantification of Doxorubicine (Dox) uptake by human CCD dermal fibroblasts at 3h (A,B) and 24h (C,D) after treatment with drug loaded nanoparticles versus free drug. Detection of Dox (A,C) was measured into the Per-CP-Cy5.5 channel. Light scattering modifications upon treatment were assessed by FSC-A/SSC-A measurements. FSC = forward scattered light; SSC - side scattered light.

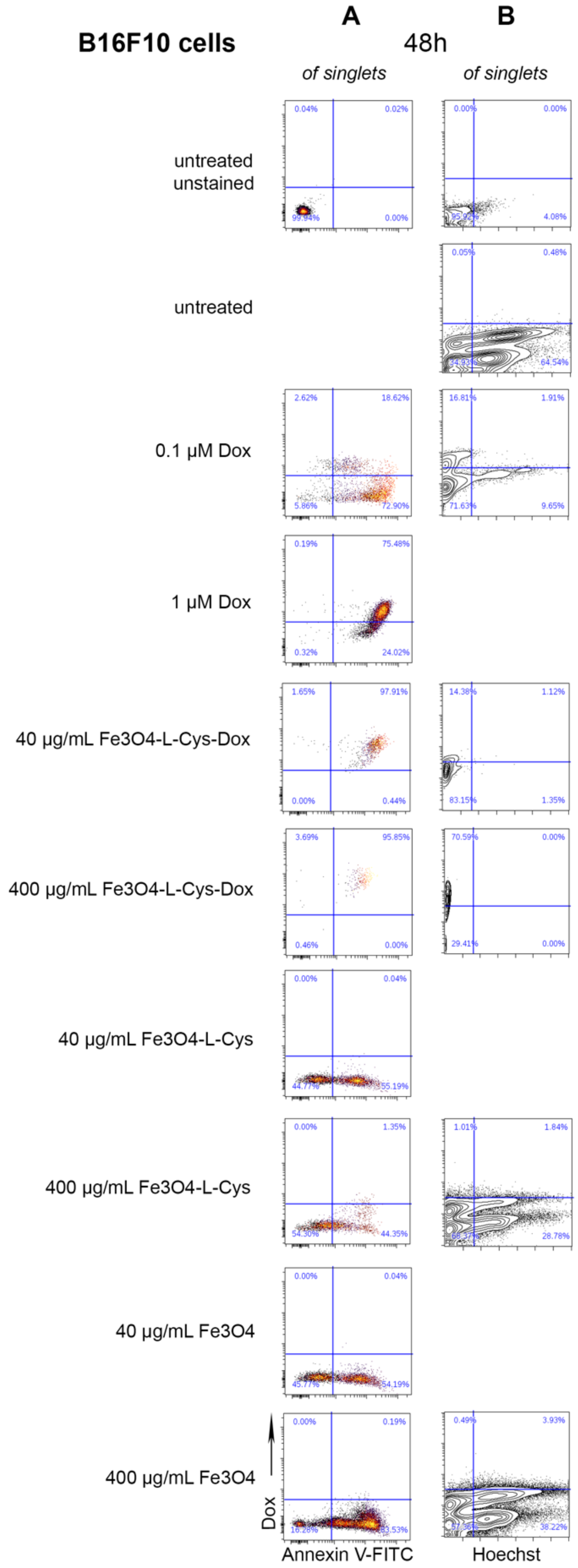

Figure S3. Cytometric evaluation of cytotoxic and cytostatic potential of Fe_3O_4, Fe_3O_4-L-Cys, and Fe_3O_4-L-Cys-Dox NPs. Analysis of the percentage of cells in apoptosis (A) and the cell cycle profile (B) following 48 h treatment with NPs or free Dox in mouse B16F10 melanoma cells.

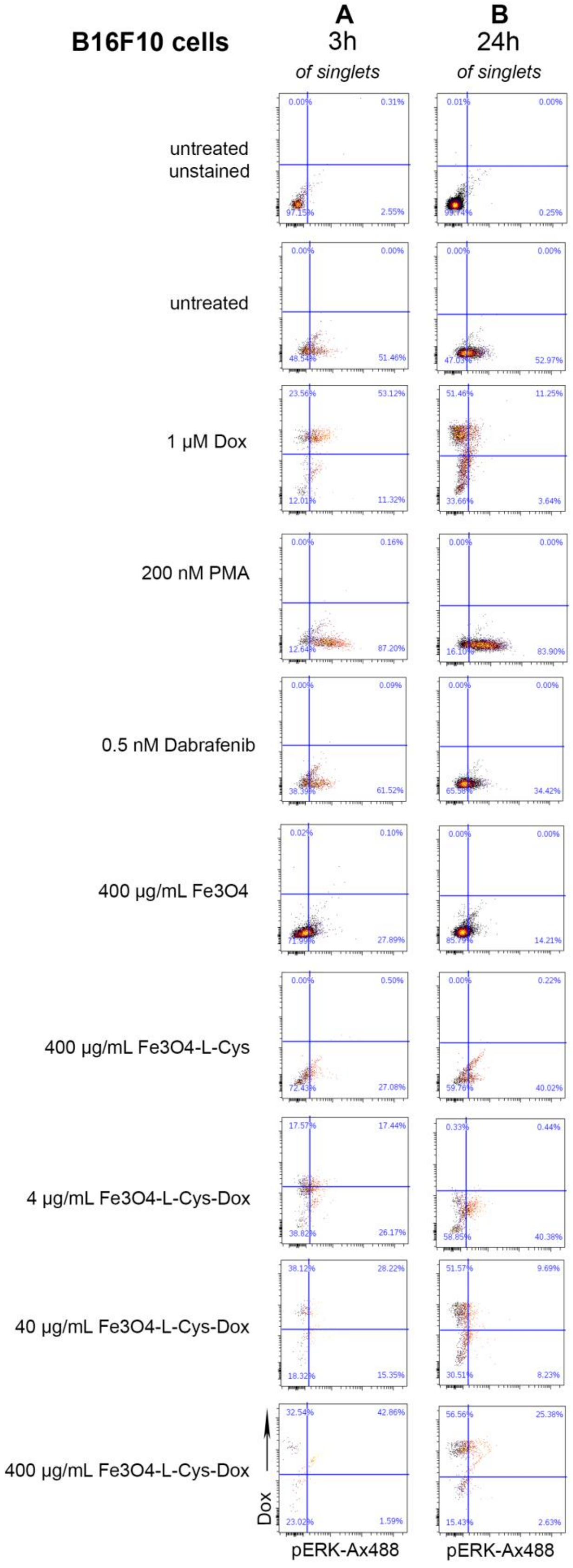

Figure S4. Cytometric evaluation of pERK expression at 3 h (A) and 24 h (B) after incubation of B16F10 cells with nanoparticles or free Dox. Phosphorylated ERK was detected on the FITC channel, upon labeling with Alexa Fluor 488-conjugated secondary antibodies, and Dox on the PerCP-Cy5.5 channel.

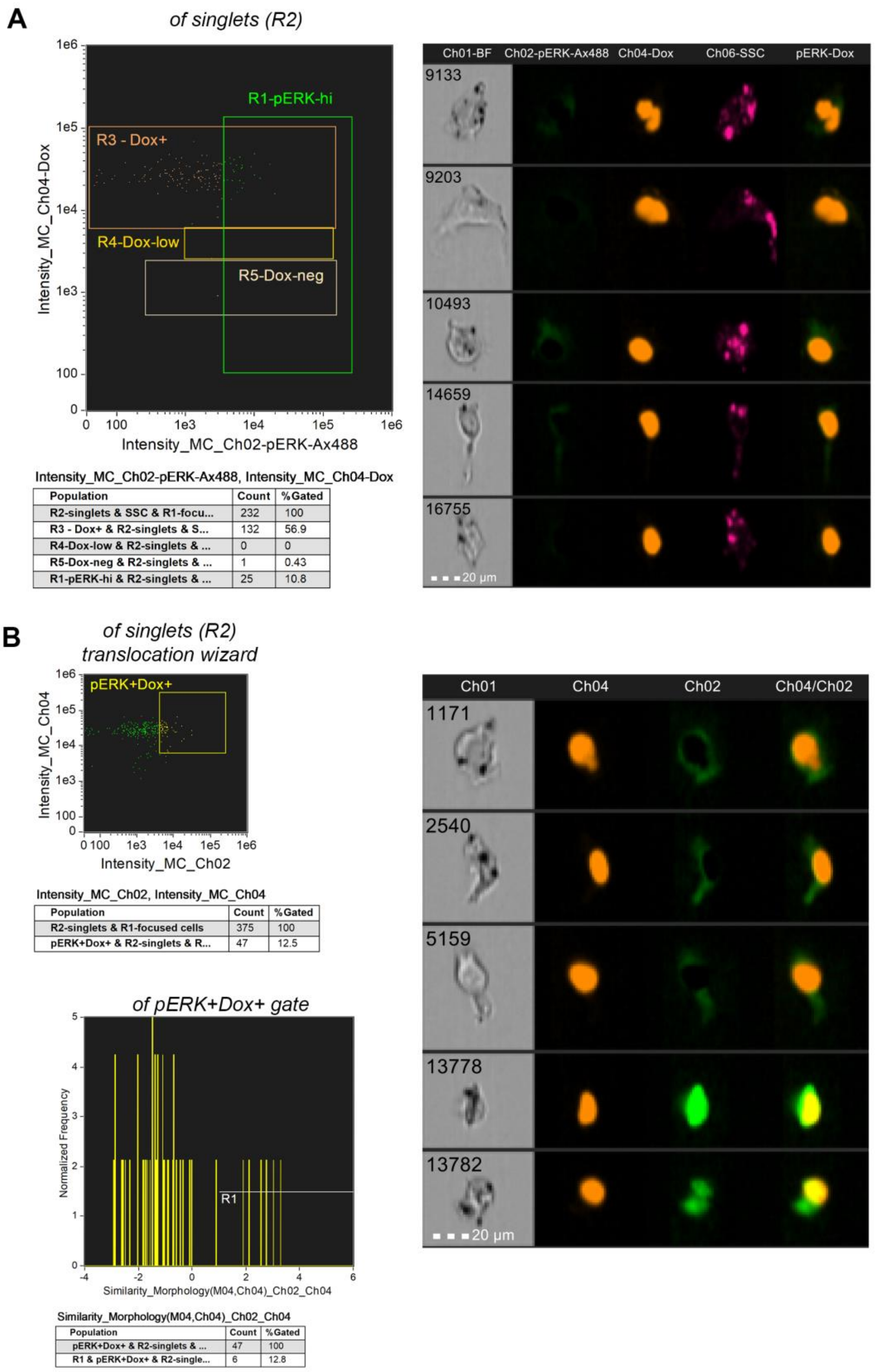

Figure S5. Imaging flow cytometry analysis of pERK expression at 3 h after incubation of B16F10 cells with 4 µg/mL Fe_3O_4-L-Cys-Dox nanoparticles. Phosphorylated ERK was detected on channel 02, and Dox on channel 04. Merged Ch02/Ch04 fluorescence, as well as single channel brightfield (Ch01) and side scatter (SSC, Ch06) images are provided. Dot plots as well as representative images of single cells are shown for each analysis. (A) R1 gate marks cells with high ERK activation, irrespective of Dox uptake level. Events from this gate are exemplified in the corresponding images. (B) Gated cells represent pERK$^+$ cells that have taken up the drug. Cells expressing pERK in the nucleus were analyzed using the translocation wizard (defined as R1). R1 events are exemplified in the two last rows of images in each data set.
